# Transfer hydrogenation of nitroarenes using cellulose filter paper-supported Pd/C by filtration as well as sealed methods†

**DOI:** 10.1039/d2ra01151d

**Published:** 2022-04-07

**Authors:** Dmitry Olegovich Bokov, Mustafa Z. Mahmoud, Gunawan Widjaja, Wanich Suksatan, Supat Chupradit, Usama S. Altimari, Hussein Ali Hussein, Yasser Fakri Mustafa, Milad kazemnejadi

**Affiliations:** Institute of Pharmacy, Sechenov First Moscow State Medical University 8 Trubetskaya St., bldg. 2 Moscow 119991 Russian Federation; Department of Radiology and Medical Imaging, College of Applied Medical Sciences, Prince Sattam bin Abdulaziz University Al-Kharj 11942 Saudi Arabia; Faculty of Health, University of Canberra Canberra ACT Australia; Postgraduate Study, Universitas Krisnadwipayana Bekasi Indonesia; Faculty of Public Health, Universitas Indonesia Depok Indonesia; Faculty of Nursing, HRH Princess Chulabhorn College of Medical Science, Chulabhorn Royal Academy Bangkok Thailand; Department of Occupational Therapy, Faculty of Associated Medical Sciences, Chiang Mai University Chiang Mai 50200 Thailand; Al-Nisour University College Baghdad Iraq; Scientific Research Center, Al-Ayen University Thi-Qar Iraq; Department of Pharmaceutical Chemistry, College of Pharmacy, University of Mosul Mosul-41001 Iraq; Department of Chemistry, College of Sciences, Shiraz University Shiraz 71946-84795 Iran miladkazemneja@yahoo.com

## Abstract

A reductive filter paper for selective nitro reduction has been prepared by modification of a pristine cellulose filter paper by Pd/C nanoparticles, as a portable catalyst. The reaction was performed in two different set-ups including (i) filtration and (ii) sealed systems, in the presence of ammonium formate and *ex situ* generated hydrogen gas reducing agents, respectively. In the sealed system in the presence of H_2_ gas, the halogenated nitroarenes were completely reduced, while in the filtration system, different derivatives of the nitroarenes were selectively reduced to aryl amines. In both systems, the reduction of nitroarenes to aryl amines was performed with high efficiency and selectivity, comparable to a heterogeneous system. Reaction parameters were comprehensively designed using Design Expert software and then studied. The properties of the catalytic filter paper were studied in detail from the points of view of swellability, shrinkage, reusability, and stability against acidic, alkaline, and oxidative reagents.

## Abstract 2

A novel and efficient catalytic filtration has been developed for the selective reduction of nitro compounds on a Pd/C-doped cellulose filter paper.

## Graphical abstract

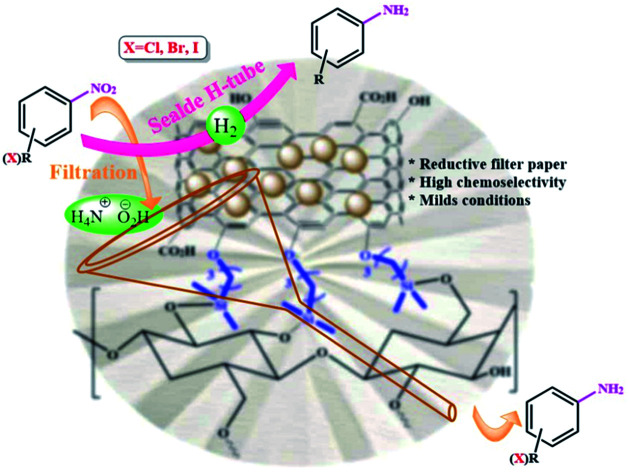

## Introduction

**Scheme 1:**
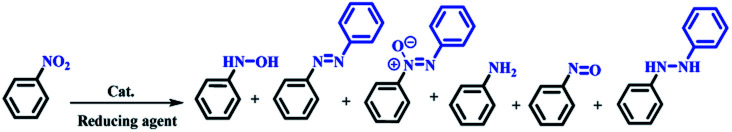
Six possible products from the reduction of nitrobenzene.^5^

The nitro group is one of the most versatile moieties in organic synthesis, mostly due to its conversion to primary and secondary amines.^1,2^ Aryl amines play vital roles in the formation of pharmaceutical and numerous biologically active molecules and are key precursors/intermediates for the synthesis of agricultural chemicals, polymers, medicines, and dyes.^3^ Kadam and Tilve reviewed the recent advancements in methodologies for the reduction of nitroarenes with differentiation of various reducing agents.^4^ The selectivity of the catalyst for transfer hydrogenation of the nitro group is an important factor from the point of view of industrial applications, environmental protection, easy work up, purity of products, and cost-effectiveness because the reduction of nitroarenes creates six different products including oxime, azo, nitroso, amine, hydrazine, and diazine oxide (Scheme 1).^5^ In addition, in multi-substituted nitroarenes containing several reducible groups such as iminium, nitrile, and olefin bonds, the selectivity of the catalyst is of high importance.

The selectivity of the catalyst is also important in reducing the nitro compounds in the homogeneous solution phase, because solvents such as ethanol and methanol in the presence of Pd can be converted to an alkylating agent (with the formation of acetaldehyde and formaldehyde) and give monoalkylated and dialkylated products.^1^ In addition, the formation of reduced by-products for the halogenated nitroarenes was another challenge that occurs by Pd/C catalyst in the presence of a reducing agent.^6^

Various catalytic systems have been reported for the transfer hydrogenation of nitro groups, including sulfide/carbides,^7,8^ nitrides and borides,^9^ metals, metal oxides,^10,11^ and functional carbon materials so far.^12^ Palladium supported on carbon (Pd/C) has a special place in organic synthesis and transfer hydrogenation of various reducible functional groups.^13^ Also, Pd/C has been used to eliminate bromate in water,^14^*N*-formylation of amines,^15^ Stille^16^ and Suzuki^17^ couplings, and aerobic esterification of alcohols.^18^

Pd/C NPs are widely used in nitro reduction in the presence of various H-sources, that could point to some examples: (1) Pd/C with ammonium formate or hydrazine hydrate,^6,19^ (2) Pd–C with polymer-supported formate,^2^ (3) Pd/C with ammonium formate/silica,^20^ (4) CoFe_2_O_4_@Pd/activated carbon nanocomposite/NaBH_4_,^21^ and (5) Pd/CNS (carbon nanospheres) under microwave irradiation.^17^ In addition, various reducing agents including hydrogen gas, NaBH_4_, silyl hydrides, hydrazine hydrate, *in situ* H_2_ generation, direct metal, MPV type redox processes using organic reducing agents (transfer hydrogenation), light induced photo catalysis (photocatalytic hydrogenation using a hole scavenger), biotic reduction, and electrocatalytic hydrogenation accompanied by water oxidation were used for transfer hydrogenation of nitro groups.^4^ Recently, Goyal *et al.* reported using MeOH as a H_2_ source for Pd/C catalyzed *N*-methylation of nitroarenes.^22,23^ Formate ion has attracted the attention of many researchers in this field due to its ease of use, safety, high selectivity, cost-effectiveness, environmental friendliness, and excellent performance. There are various reports for the application of formate to transform hydrogenation of various functional groups, especially in the form of polymer supported.^2,3,24^

In addition, a green way to perform transfer hydrogenation under a H_2_ atmosphere is the *ex situ* production of H_2_ in a sealed tube by the reaction of an acid with a metal. The Skrydstrup group developed a two-chamber H-tube reactor to perform the reactions under *ex situ* generated gases.^25^ Carbonylation,^26^ hydrogenation of unsaturated bonds,^27^ preparation of vinyl arenes,^28^ and arene cyanation^29^ were some of the reactions developed by the Skrydstrup group *via ex situ* generated CO_2_, H_2_, ethylene, and HCN gases, respectively. In this paper, we will show the successful performance of this system in the reduction of nitro groups in the presence of *ex situ* generated H_2_ gas using a novel catalyst.

Although heterogeneous catalysts have advantages such as easy work-up, recoverability, *etc.*, their catalytic activity is lost during successive cycles.^30^ In addition, contamination with raw materials requires frequent and tedious washing, as well as incomplete recovery from the reaction medium which are some of the drawbacks associated with heterogeneous catalysts, which causes their catalytic activity to decrease over time, and limits their application in the case of very small nanoparticles such as Pd/C. In this way, developing green approaches is now a mandatory prerequisite for the synthetic organic chemists.

A smart strategy to address the mentioned drawbacks associated with the heterogeneous systems is to design a portable system by immobilizing the small nanoparticles on a filter paper. Numerous reports of manipulation and use of filter paper as a catalyst in various reactions are available. The use of filter paper in organic synthesis is an intelligent strategy for solid phase synthesis that has advantages such as clean work-up.^31^ Due to the high ability to manipulate and modify a cellulose paper, it has attracted a lot of attention from scientists in various fields in order to achieve different goals.^32^ In the field of organic chemistry, modifications have been used to convert cellulose paper into a catalyst as a suitable substrate. Cellulose has been known as a powerful solid support in organic synthesis.^33^ Previously, a modified porous microstructure-cellulose paper by methacryloxy groups (*via* a silane coupling method) was used as a support for the immobilization of lipase enzymes (due to hydrophobic interaction). The resulting paper showed noteworthy catalytic activity towards nonaqueous transesterification reaction in both batch and flow modes by immersion of the modified FP in the solution.^34^ Ag NP-loaded filter paper was developed by Mourya *et al.* as a catalytic system for nitro reduction, cascade reaction, and degradation of methyl orange, which was prepared by dipping the FP into hydrophobic monodispersed AgNO_3_ solution.^35^ Also, AgNPs, AuNPs, and Ag–Au bimetallic nano-alloy deposited onto a cellulose solid support were used for catalytic reduction of *p*-nitroaniline.^36^ Cellulose paper is also a suitable substrate for the *in situ* preparation of nanoparticles. For example, Ni^37,38^ or Co^39^ nanoparticles were prepared by electrostatic interactions in cellulose paper coated with a thin layer of chitosan. For this purpose, metal ions were adsorbed on chitosan-coated filter paper and then reduced by NaBH_4_ solution and finally metallic nanoparticles are formed.^37–39^

In another report, a 3-mercapto-propanoic acid-modified cellulose filter was utilized as an adsorbent for the efficient removal of arsenate from drinking water through a batch or semi-continuous membrane separation process.^40^ A dispersion of Pd NPs in ammonium salts of polystyrenes could be efficiently immobilized on the surface of a filter paper using heating along with dicarboxylic acid or halide anions. By dipping one piece of the resulting modified filter paper to the reaction mixture, it was efficiently used for sequential cross-coupling and hydrogenation reactions.^31^ The electrostatic interaction between ionic ammonium groups in Pd@HPS-NR_3_^+^Cl^−^ (HPS = hyperbranched polystyrene) NPs with surface hydroxyl groups in cellulose was responsible for this strong immobilization.^31^ Wei *et al.* developed an Fe-tannin-framework ink coating to prepare a cellulose-derived catalyst using commercial cellulose filter paper as a carbon source. The resultant Fe_3_C/Fe–N–C catalyst was used for oxygen reduction under alkaline conditions.^41^ Moreover, the Au NP-loaded filter paper composite presents high surface enhanced Raman (SERS) efficiency, which provides real-time monitoring of chemical reactions.^42^ The paper was fabricated by the dip coating process using concentrated gold NP suspensions in toluene. Superhydrophobic paper with high self-cleaning properties,^43^ paper-based electrodes,^42^ Ag-doped cellulose filter paper as a wound dressing agent,^44^ and superhydrophobic cellulose paper for water drop energy harvesting^32^ are some recent advances over manipulation of cellulose filter paper and its application in various fields of science.

**Scheme 2:**
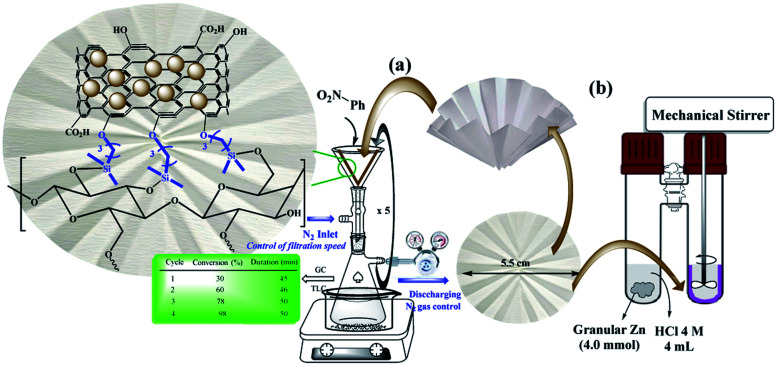
Two setups for the (a) selective reduction of nitroarenes by the filtration method (method A), and (b) complete reduction of halogenated nitroarenes *via* the *ex situ* generated H_2_ gas in a sealed tube (method B) on FP@Si@Pd/C catalytic filter paper.

Despite advances in this field, the use of modified filter papers by reaction mixture filtration has not been studied. The main goal of the present work was the introduction of “potable catalysts” based on cellulose filter paper *via* filtration of the reactants. In this way, the cellulose filter paper was modified with Pd/C nanoparticles that its application in reduction of a variety of substrates and functional groups in the presence of ammonium formate is well known.^2,6,17,19–21^ In this work, Pd/C nanoparticles were immobilized on a pre-silylated filter paper. Ammonium formate was used as the H-source in the reaction mixture (Scheme 2).

Our studies on the resulting catalytic filter paper also showed that in the presence of *ex situ* generated H_2_ gas in a sealed two-chamber H-tube system, the halogenated nitroarenes were reduced entirely. Therefore, by changing the reducing agent, the filter paper has the ability to selectively reduce different functional groups.

## Results and discussion

**Scheme 3:**
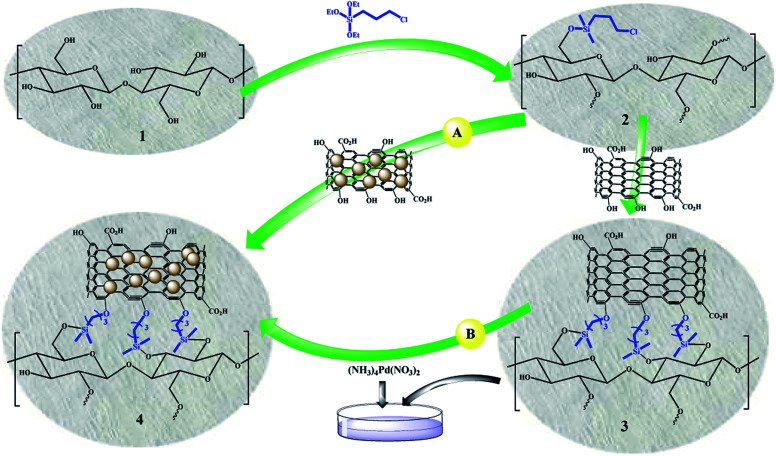
General schematic view for the preparation of FP@Si@Pd/C catalytic filter paper (4) *via* two different routes.

Scheme 3 shows two different routes for the preparation of FP@Si@Pd/C catalytic filter paper. Given that Pd interacts strongly with oxygen atoms,^45^ both methods are highly reliable for immobilizing Pd/C on the cellulose filter paper. In route a, the pre-prepared Pd/C was immobilized in one step on the silylated cellulose filter paper. Route b consists of two steps of immobilizing activated carbon on the silylated filter paper and then immobilizing Pd on the activated carbon. Although both methods can be used for the preparation of the catalytic filter paper, in this paper, route a was used for the sake of easier workup, greater repeatability, and a higher Pd loading percentage.

### Paper characterization

The catalytic filter paper 4 was also characterized by different analyses at each stage, and after confirmation, the synthesis steps were continued. Filter papers 2–4 as well as unmodified (pristine) filter paper were characterized by the ATR-IR technique (ESI, Fig. S1†). The presence of strong vibrations at 780 cm^−1^ and 1073 cm^−1^ was related to the symmetric and asymmetric Si–O–Si stretching vibrations, respectively,^46^ which confirms the successful silylation of the filter paper. The immobilization of activated carbon on the silylated filter paper was also confirmed by the presence of the characteristic peaks related to CO_2_H (carboxylic acid) as well as strong vibrations of C=C (corresponding to benzene rings) at 1760 cm^−1^ and 1440–1640 cm^−1^ in filter paper 3, respectively (Fig. S1-e†).

Although several methods have been used to silylate cellulose, silylation of a cellulose filter paper has not been specifically reported. In this paper, inspired by previously reported methods on cellulose,^46,47^ the cellulose filter paper was successfully silylated with a high wt% Si. Silylation of a filter paper was performed using both aqueous solution and chemical vapor deposition (CVD) methods and studied by EDX (mean of 5 points) as well as ICP analyses. ICP analysis was performed after preparation of ash from the filter paper at 500 °C. According to the results, % wt Si in the silylated filter paper by aqueous solution and CVD was equal to 14.68% wt and 12.90% wt respectively by ICP analysis, respectively (Table 1). As shown in Table 1, the results of ICP and EDX were so close to each other, which confirms the accuracy of the results and the subsequent silylation successfully. Despite the similarity of the results of both CVD and immersion method (solution), the filter paper was silylated by the immersion method due to advantages such as room temperature, simple work up, cost-effectiveness, *etc.* In the CVD method, different exposure times of 2, 6, 8, and 12 h were studied, among which the highest (3-chloropropyl)triethoxysilane (CPTES) loading occurred at 6 hours and the loading rate did not increase significantly after that. The loading rates at the exposure times of 2, 6, 8, and 12 h were equal to 7.14, 12.77, 12.76 and 12.80% wt, respectively. It seems that after 6 hours the surface becomes saturated and cannot be loaded more by CPTES.

**Table 1:** Elemental analyses of the silylated filter paper resulting from ICP as well as EDX analyses

Element	CFP		SiCFP_(aq)_^a^		SiCFP_(vap)_^b^	
	ICP^c^ (wt%)	EDX^d^ (wt%)	ICP^c^ (wt%)	EDX^d^ (wt%)	ICP^c^ (wt%)	EDX^d^ (wt%)
C	—	51.36	—	48.60	—	48.09
O	—	48.64	—	34.96	—	34.18
Si	—	—	14.68	11.36	12.90	11.96
Cl	—	0.00	—	5.08	—	5.77

a Cellulose filter paper silylated from aqueous solution of CPTES as a soaking method.

b Cellulose filter paper silylated from the chemical vapor deposition method for 6 h time interval.

c Resulted from ash of the silylated filter paper.

d Mean of 5 points.

In the next step, in order to ensure the successful immobilization of Pd/C on the filter paper, the filter paper was placed on a glass funnel and washed 3 times, each time with 10 mL of absolute ethanol, and the remaining solution (filtrate) was analyzed by ICP-MS analysis. No trace of any metal (including Pd and Si) was observed in the results, which reflects the stable immobilization of the activated carbon through the ether bond according to what is shown in Scheme 3. The results of ICP analysis showed that 9.45% wt Pd was loaded on one filter paper with a diameter of 5.5 cm and a weight of about 120 g m^−2^.

**Fig. 1:**
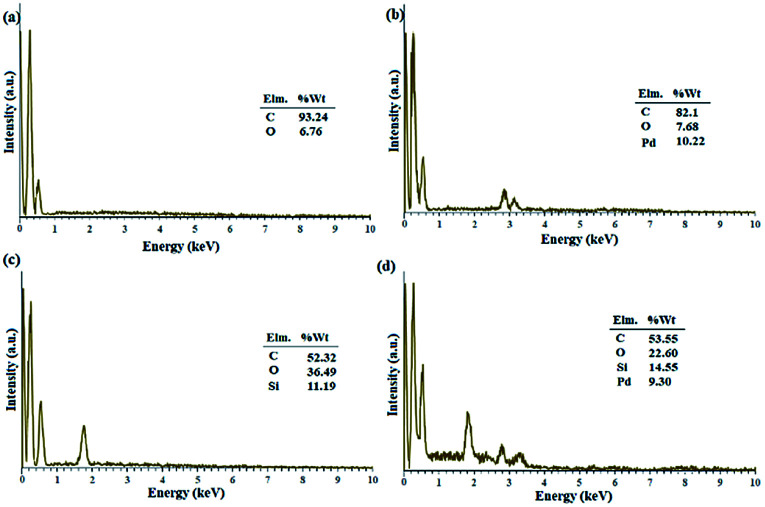
EDX spectra of (a) activated carbon, (b) Pd on activated carbon (Pd/C), (c) activated carbon-immobilized cellulose filter paper (FP@C), and (d) Pd on activated carbon-immobilized cellulose filter paper (FP@Si@Pd/C) prepared *via* route b presented in Scheme 3.

Fig. 1 shows the EDX analysis of various steps in the synthesis of the catalytic filter paper. The results showed high purity of the filter paper and its successful silylation with 11.36 wt% Si (ESI, Fig. S2-b†). The presence of oxygen in the EDX spectrum of the activated carbon indicates the hydroxyl and carboxylic acid functions in the structure of activated carbon, completely consistent with the FTIR spectrum of the activated carbon (ESI, Fig. S1†).

Fig. 1b shows the preparation of Pd/C with 10.22 wt% Pd. No impurities due to the presence of other elements in the Pd/C structure were observed according to the corresponding EDX spectrum. Fig. 1c shows the immobilization of the activated carbon on the filter paper (route b, Scheme 3). Successful silylation of the filter paper as well as Pd/C led to its subsequent successful immobilization on the filter paper in route b (Scheme 3).

**Fig. 2:**
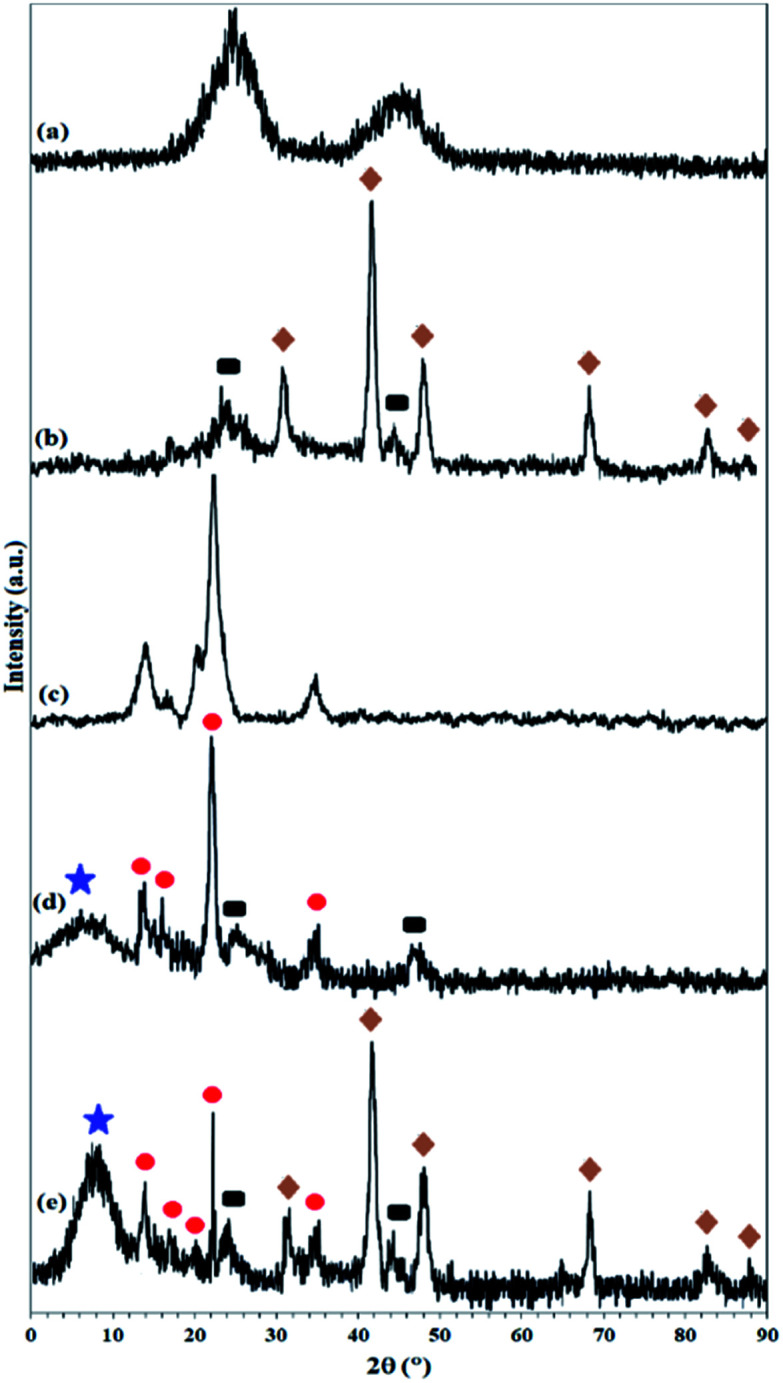
XRD pattern of (a) activated carbon, (b) Pd on activated carbon (Pd/C), (c) pristine cellulose filter paper, (d) activated carbon-immobilized cellulose filter paper (FP@C), and (e) Pd on activated carbon-immobilized cellulose filter paper (FP@Si@Pd/C). Symbol representation: rectangle: carbon phases; star: silica phases; circle: cellulose phases; rhombic: Pd NP phases.

As shown in Fig. 1, 9.3% wt Pd was loaded on a 5.5 cm diameter filter paper (Fig. 1d). The X-ray diffraction pattern of the filter paper at different stages of the preparation confirms the modifications at each stage, in full agreement with EDX and FTIR analyses (Fig. 2). Activated carbon was characterized by two peaks at 2*θ* = 24.0° and 44.1° with an amorphous structure, in agreement with the literature (Fig. 2a).^48^ The Pd crystalline pattern was detected by five characteristic peaks at 2*θ* = 41.9°, 48.0°, 68.1°, 82.2°, and 88.1° corresponding to (111), (200), (220), (311), and (222) planes respectively, completely in agreement with the reported Pd NP crystal structure (Fig. 2b).^45^ Reduction of AC corresponding peak intensity was a strong proof for the successful supporting of Pd on the activated carbon. The mean particle size of the Pd/C NPs was calculated from the Pd(111) peak using Scherrer's equation:^49^
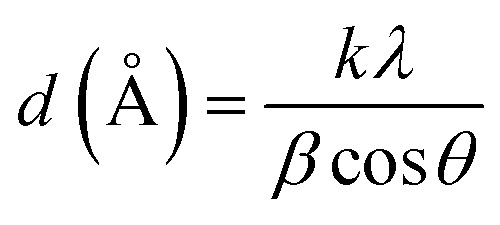
where *k* is a constant equal to 0.9, *λ* is the wavelength of the X-ray used (1.54056 Å), *β* is the broadening of the diffraction line measured at half of its maximum intensity in radius, and *θ* is the Bragg diffraction angle (angle at position of peak maximum). According to Scherrer's equation, the average particle size of Pd/C NPs was calculated to be 2.6 nm.

The presence of peaks appearing at 2*θ* = 14.1°, 16.2°, and 22.2° indicates the crystalline structure of the cellulose filter paper, in full accordance with the literature (Fig. 2c).^39^ The X-ray diffraction pattern resulting from the immobilization of amorphous activated carbon on the cellulose paper shows the presence of both carbon and cellulose phases with the corresponding peaks, which reflects the lack of structural deviation of cellulose as well as the activated carbon. The presence of an amorphous peak at 2*θ* = 7.9° corresponds to amorphous silica immobilized on the cellulose fibers (Fig. 2c).^50^ The X-ray diffraction pattern in the final filter paper also includes the presence of all phases of carbon, cellulose, silica and Pd NP, which confirms the success immobilization of all groups on the cellulose surface (Fig. 2d).

**Fig. 3:**
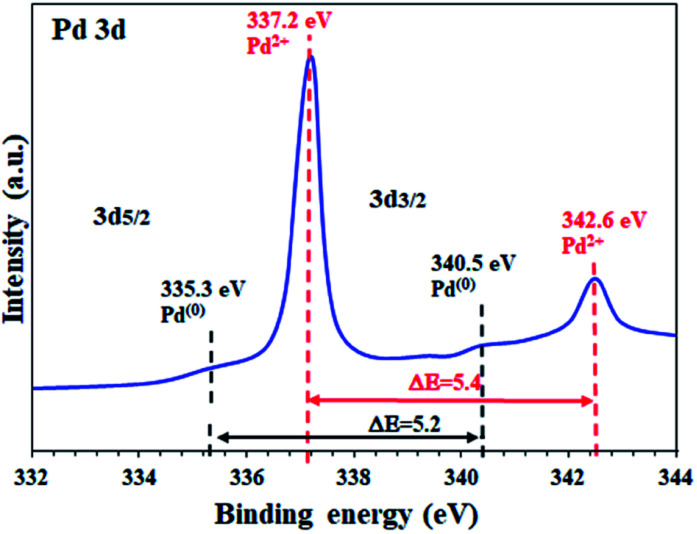
High resolution XPS analysis (energy corrected, normalized) of Pd on activated carbon-immobilized cellulose filter paper (FP@Si@Pd/C).

To determine the oxidation state of the Pd sites supported on the activated carbon in FP@Si@Pd/C, high resolution Pd 3d XPS analysis was performed. As shown in Fig. 3 two characteristic peaks at 337.2 eV and 342.6 eV were assigned to Pd 3d_5/2_ and Pd 3d_3/2_ related to Pd^2+^ respectively, in agreement with the literature.^5^ Also, the energy band gap equal to Δ*E* = 5.4 eV confirms the oxidation state of +2 for the Pd sites.^2,5,19^ Based on the previous reports, Pd supported on activated carbon contains a mixture of Pd^2+^ as well as Pd^(0)^, mostly Pd^2+^.^51,52^

The as-prepared Pd/C NPs were also studied by XPS analysis in the Pd-3d range binding energies. The results showed a mixture of Pd(0) and Pd(+2) oxidation states for Pd/C, in full agreement with the previous reports (ESI, Fig. S3†).^51,52^ As shown in the figure, the Pd centers were mostly in the zero-oxidation state, which, after immobilization on the silylated filter paper, oxidize to the +2 state mostly (Fig. 3). According to the observations and studies on the NaBH_4_-mediated preparation of Pd/C NPs, two reasons can be given for the mixture of Pd(0) and Pd(+2) oxidation states in Pd/C: (1) in these methods, Pd^2+^ salts (Pd(OAc)_2_, PdCl_2_, *etc.*) were used to coordinate Pd on AC, so all Pd centers are initially Pd^2+^, and (2) considering that a mixture of Pd^2+^ and Pd^0^ oxidation states was observed in FP@Si@Pd/C, it seems that the immobilization of Pd/C on the silylated cellulose filter paper caused surface oxidation due to air exposure after reduction.

The high resolution Pd 3D XPS spectrum of FP@Si@Pd/C also shows the Pd^(0)^ sites with the corresponding binding energies at 335.3 eV and 340.5 eV with an energy band gap of Δ*E* = 5.2 eV, in agreement with the literature.^2,19^

**Fig. 4:**
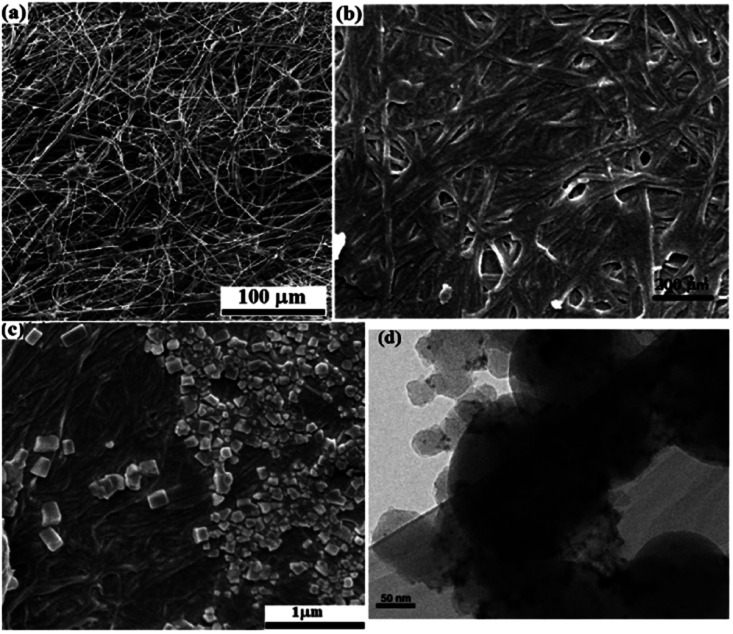
FESEM images of (a) pristine cellulose filter paper, (b) silylated FP, and (c) FP@Si@Pd/C. (d) TEM image of the separately prepared Pd/C.

SEM images from the plain, silylated and modified filter paper with Pd/C also show well the preparation of filter paper 4, in accordance with other analyses. As shown in Fig. 4a, the cellulose fibers in the plain filter paper has a diameter of about 17 μm, which after silylation reaches about 22 μm (Fig. 4b). In addition, the density of the cellulose fibers was slightly increased and the amount and diameter of the porosities were reduced. Also, the plate-shaped areas (non-fibrous areas) represent the cross-linked cellulose fibers resulting through silica groups. Immobilization of Pd/C on the silylated cellulose fibers results in clearer cubes (related to Pd/C sites) in the FE-SEM image of FP@Si@Pd/C, indicating the successful immobilization of Pd/C on the cellulose fibers (Fig. 4c). Fig. 4d shows the separately prepared Pd/C with an average diameter of 3.5 nm. No considerable agglomeration was found in the TEM image of the Pd/C, reflecting its stability and high dispersion ability.

The porosity and surface area of the plain and modified filter papers were studied by the BET method. The results show that the Pd/C was successfully immobilized on the filter paper. According to the results of BET presented in Table 2, the plain filter paper has a specific surface area and a pore size of 1.5 m^2^ g^−1^ and 8.64 μm respectively, which after silylation reach 1.68 m^2^ g^−1^ and 6.45 μm. Unlike nanoscale compounds, increasing the surface area of bulk particles indicates functionalization.^53,54^ Immobilization of the AC causes a significant increase in specific surface area up to 3.75 m^2^ g^−1^ and a significant decrease in pore size up to 1.54 μm. Pd/C immobilization on the cellulose fibers provides a smaller surface area compared to AC immobilization on the cellulose, indicating successful Pd loading on carbon. The average pore size was almost the same as before and was equal to 1.55 μm (Table 2, entry 4).

**Table 2:** Surface characteristics of the plain cellulose FP, FP@Si–Cl, FP@C, and FP@Si@Pd/C

Entry	Sample	Specific surface area (m^2^ g^−1^)	Average pore size (μm)
1	Plain cellulose FP	1.55	8.64
2	FP@Si–Cl	1.68	6.45
3	FP@C	3.75	1.54
4	FP@Si@Pd/C	3.05	1.55

Given that the nitro reduction will be done by the filtration setup, the amount of swelling of the filter paper by the reaction solvent is a critical parameter. Accordingly, the most suitable solvent is that preferably has the highest swelling rate and also provides satisfactory efficiency for the nitro reduction. Table 3 shows the results of swelling measurements for the plain, silylated, FP@Si@C, and FP@Si@Pd/C filter papers.

**Table 3:** Swelling measurements of the plain, silylated, FP@C, and FP@Si@Pd/C

Solvent	Swelling (g mL^−1^) of filter paper			
	Plain	Silylated	FP@C	FP@Si@Pd/C
H_2_O	8.8	10.0	14.5	14.0
H_2_O : MeOH	11.2	15.2	19.8	19.1
DMF	13.3	14.0	19.0	17.6
EtOH	6.8	9.1	14.5	13.5
MeOH	7.5	9.6	15.2	13.7
DMSO	14.2	15.7	18.8	17.2
Toluene	0.6	0.6	1.9	1.0
Acetic acid	5.9	7.2	8.7	8.2
Acetone	1.5	3.0	4.5	4.4

Silylation of the surface of the filter paper significantly increased the swelling, which was higher for protic solvents than for polar ones. Possible cross-linking between the cellulose fibers results in a rigid structure and greater stability than in the plain filter paper. Cellulose fibers have intermolecular- and intramolecular H-bonding interactions. By silylation of these chains, many of these hydrogen bonds are broken, providing an intermolecular hydrogen bond with the solvent molecules and consequently increasing the swelling rate in the protic solvents. As shown in Table 3, the amount of swelling in polar solvents such as DMSO and DMF has decreased compared to the amount in the silylated filter paper; however, for protic solvents, the reduction in swelling was slightly lower than for the silylated filter paper. The highest swelling was measured for FP@Si@Pd/C in H_2_O : MeOH equal to 19.1 mL g^−1^.

### Optimization studies over the reaction parameters

**Fig. 5:**
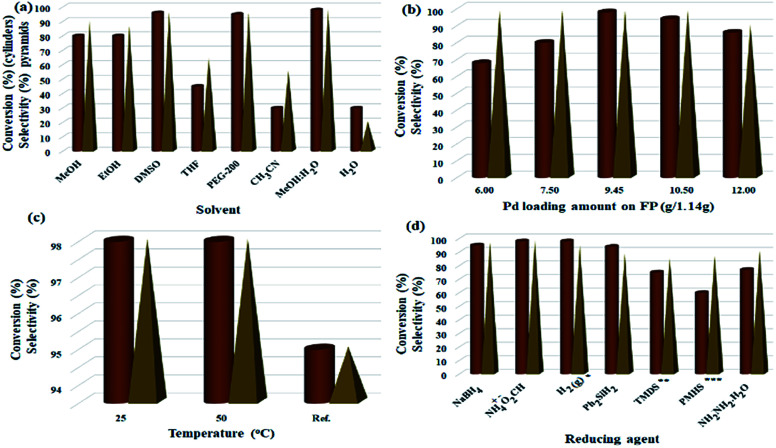
Optimization of parameters of nitrobenzene reduction to aniline in method A. (a) Solvent effect (5.0 mL, FP4 9.45 wt% Pd loading, ammonium formate (10.0 mmol), R.T.), (b) Pd loading amount (g/1.14 g, MeOH : H_2_O (1 : 1, 5.0 mL), ammonium formate (10.0 mmol), R.T.), (c) solvent temperature (FP4 9.45 wt% Pd loading, MeOH : H_2_O (1 : 1, 5.0 mL), ammonium formate (10.0 mmol)), (d) reducing agent (10.0 mmol, FP4 9.45 wt% Pd loading, MeOH : H_2_O (1 : 1, 5.0 mL), R.T.); to study the temperature effect, the reaction set-up was performed on a water bath. * Heating was applied on a temperature-controlled water bath. **H_2_ gas was applied to the system according to method B presented in Scheme 2, ***1,1,3,3-tetramethyldisiloxane (TMDS); ****polymethylhydrosiloxane (PMHS).

Parameters including Pd/C loading on one filter paper, reaction temperature, and number of filtrations were designed and studied by Design Expert software (ESI, Fig. S4–S6, and Tables S1–S3†). Fig. 5 shows some of the tests performed in accordance with the designed experiments by Design Expert software. Examination of the effect of different solvents showed that DMSO, MeOH : H_2_O mixture, and PEG-200 had the highest efficiencies (96%, 98% and 95%, respectively) and selectivity between the solvents tested. Due to the presence of several OH groups as well as polar silica groups in the cellulose fibers, it causes stronger interactions between protic and polar solvents with the cellulose fibers and consequently their better diffusion in the filter paper. CH_3_CN, THF and H_2_O had the lowest efficiency among the studied solvents (Fig. 5a). The amount of Pd loaded on the surface of the filter paper was also an effective parameter on the reduction of nitrobenzene. For this purpose, different amounts of Pd/C were immobilized on the filter paper, and then the nitrobenzene reduction efficiency was studied. The highest efficiency was obtained at 9.45% wt Pd on a filter paper, and at higher and lower values, the efficiency decreased (Fig. 5b). Also, the highest efficiency was obtained at room temperature, and at reflux temperature, the efficiency decreased slightly (Fig. 5c). H_2_ gas and ammonium formate as hydrogen sources produced the highest possible efficiency (Fig. 5d). In this study, H_2_ gas was generated *ex situ* by the reaction of Zn with HCl in a completely different set-up (Scheme 2). The effect of other reducing agents is shown in Fig. 5d.

After finding the best conditions to reduce nitro compounds, different derivatives of aryl nitro were reduced by both filtration and sealed-tube methods. Table 4 shows the results of the filtration method. The remarkable point was the selective reduction of the nitro group in the presence of substitutions such as OMe (Table 4, entry 2), OH (Table 4, entry 3), halogen (Table 4, entries 5–7), and CN (Table 4, entry 8). Also, the selectivity for all derivatives was calculated between 92% and 99%. As shown in Table 4, most of the derivatives reached the highest possible efficiency during 4 consecutive filtrations. Electronic effects showed that the presence of electron withdrawing substitutions in aryl nitro improves the reduction efficiency. This effect was observed on 6h and 6j–6l products. As will be shown in mechanistic studies, this effect can be attributed to the presence of several anionic intermediates in the reduction mechanism.

**Table 4:** Reduction of nitroarenes over FP@Si@Pd/C catalytic filter paper 4 in the presence of ammonium formate^a^

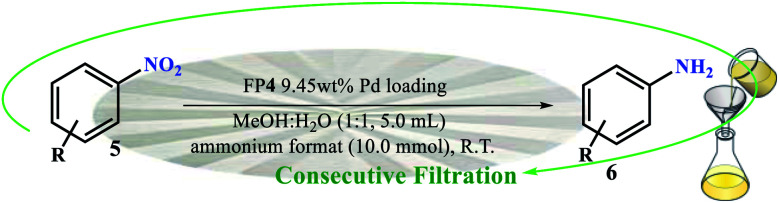					
Entry	R	Product	Time (min)/cycles	Con.^b^ (%)	Sel.^c^ (%)
1	H	6a	190/4	98	99
2	4-MeO	6b	190/4	95	98
3	4-OH	6c	190/4	78	92
4	4-Me	6d	190/4	94	99
5	4-Cl	6e	190/4	85	94
6	4-Br	6f	190/4	85	96
7	4-I	6g	190/4	95	98
8^d^	4-CN	6h	140/3	98	95
9	2-Pyridyl	6i	190/4	94	99
10	3-NO_2_	6j	140/3	95	97
11	4-COMe	6k	140/3	99	96
12	4-CO_2_Me	6l	140/3	98	96
13	3-NH_2_-4-Cl	6m	245/5	94	95

a Reaction conditions: Ar–NO_2_ (1.0 mmol), FP4 9.45 wt% Pd loading, MeOH : H_2_O (1 : 1, 5.0 mL), ammonium formate (10.0 mmol), R.T., consecutive filtration of the mixture.

b Conversion based on GC analysis.

c Selectivity towards the preparation of aniline derivatives.

d No reduction for the cyano group (4-(aminomethyl)aniline) was observed.

Next, the reduction of nitro compounds was also studied in the presence of H_2_ gas in a sealed system (Scheme 2). The results are summarized in Table 5. A noteworthy point in this study was the simultaneous reduction of halogen substitution in halogenated nitroarenes, despite the fact that the H_2_ gas pressure also appears to be low in this system. On the other hand, the reduction of nitrorenes bearing nitrile (7f), hydroxyl (7c), and MeO (7b) was done quite selectively as the filtration method. The results gave high to excellent efficiency for all derivatives, both electron donor and withdrawing groups.

**Table 5:** Reduction of nitroarenes over FP@Si@Pd/C catalytic filter paper 4 in the presence of *ex situ* H_2_ gas generated^a^

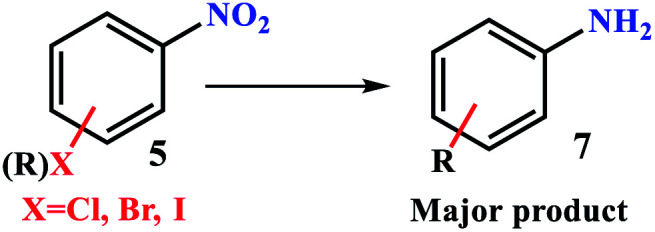					
Entry	(R)X	P.	Time (h)	Con.^b^ (%)	Sel. (%)
1	H	7a	8	96	98
2	4-MeO	7b	9	94	98
3	4-OH	7c	9	75	96
4	4-Me	7d	8	90	99
5^c^	4-Cl	7e	7	92	98
6^c^	4-Br	7e	7	90	97
7^c^	4-I	7e	7	90	97
8^d^	4-CN	7f	6.5	95	96
9	2-Pyridyl	7g	6.5	94	99
10^e^	3-NO_2_	7h	5	98	97
11	4-COMe	7i	6	96	96
12	4-CO_2_Me	7j	6	97	96
13	3-NH_2_-4-Cl	7k	7	94	98

a Reaction conditions: in a COware, H-tube^26^ at room temperature, one-side: Ar–NO_2_ (1.0 mmol), FP4 9.45 wt% Pd loading MeOH : H_2_O (1 : 1, 5.0 mL); one-side: granular Zn (4.0 mmol), HCl 4 M (4.0 mL).

b Conversion based on GC analysis.

c Aniline was prepared selectively (complete reduction of nitro and halide groups).

d 4-Aminobenzonitrile was the major product.

e 1,3-Diaminophenol was the major product.

### Chemoselectivity

In order to study more accurately and extensively the selectivity of the filter paper towards the reduction of nitroarenes, the chemoselectivity in both filtration and sealed tube methods on different di-substituent derivatives was studied. As shown in Table 6, the reduction selectivity of different nitrorenes with different reducible substitutions including nitrile, olefin, halogen, oxime, and hydroxyl was studied by both methods. In general, in the filtration method in the presence of ammonium formate as a reducing agent, a high selectivity was observed for the reduction of the nitro group in the presence of the mentioned reducible substituents, and the nitro group can be selectively reduced to amine without interference of CN, OH, olefin, halogen and oxime functional groups. The results also showed that under these conditions, the CN functional group was also selectively reduced to 1°-amine in the presence of olefin (Table 6, g), oxime (Table 6, h), and hydroxyl functional groups (Table 6, f). However, in the presence of H_2_ gas in the sealed tube method, it was shown that the catalytic filter paper has the ability to reduce groups including nitrile, olefin, halogen, and oxime, and among them, acts selectively for nitrile, olefin, halogen, and hydroxyl groups. As shown in Table 6, in the presence of the oxime functional group in 4-((hydroxyimino) methyl) benzonitrile (Table 6, h), the selectivity was lost and a mixture of reduced products containing (h_1_) and (h_2_) was obtained. The filter paper under a H_2_ atmosphere has a selectivity of X = NO_2_> => CN > N = –OH. As a result, by changing the reducing agent, a wide range of products can be selectively achieved from the reduction of nitro compounds (in the filtration method) or the reduction of aryl nitrile, styrene derivatives, and aryl halides (in the sealed method in the presence of H_2_ gas).

**Table 6:** Chemoselectivity behavior of FP@Si@Pd/C catalytic filter paper in method A and method B towards the reduction of various functional grorups^a^

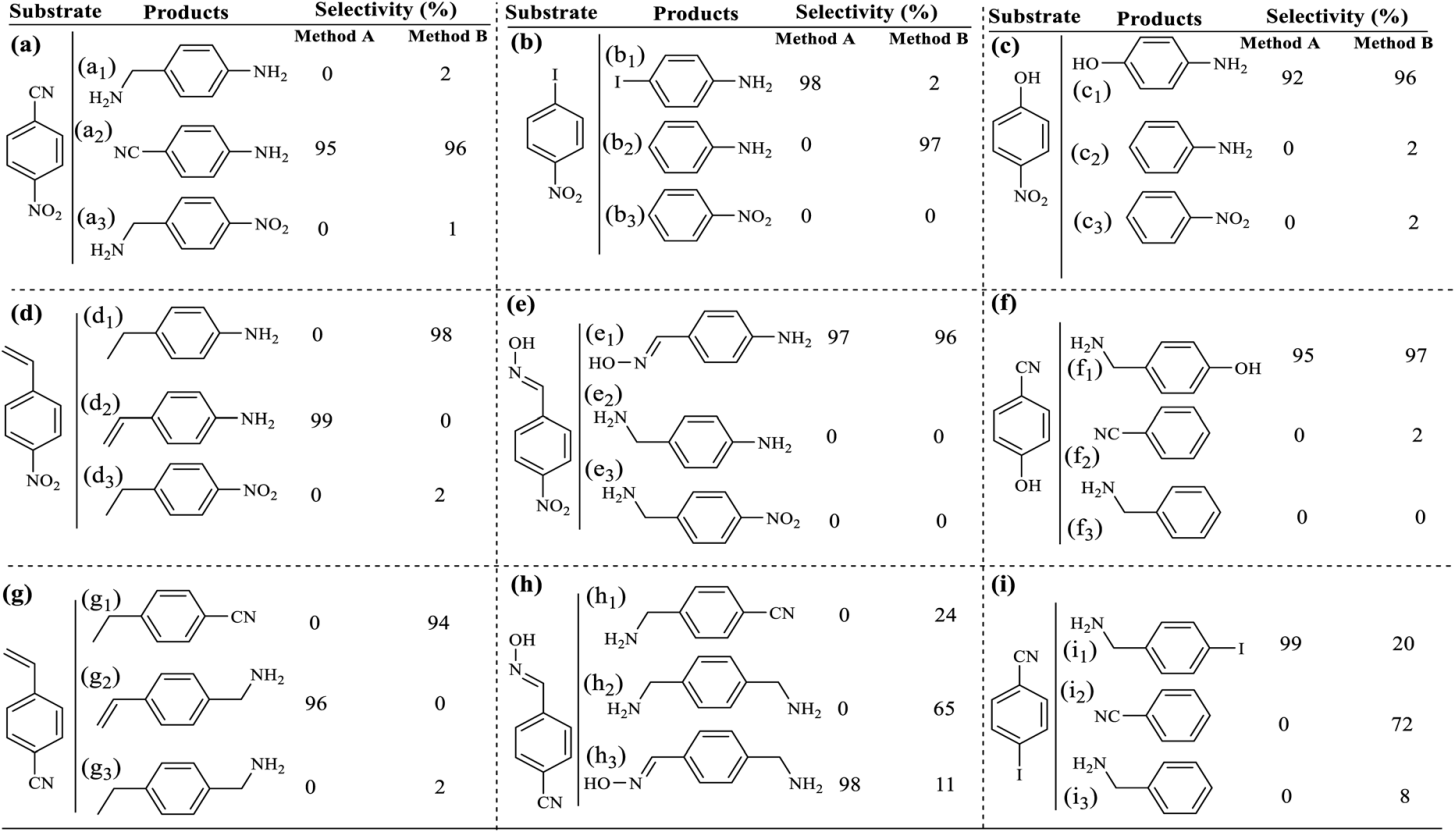

a Reaction conditions: method A: substrate (1.0 mmol), FP4 9.45 wt% Pd loading, MeOH : H_2_O (1 : 1, 5.0 mL), ammonium formate (10.0 mmol), R.T., 190 min (4 consecutive filtration of the mixture). Method B: In a COware, H-tube^26^ at room temperature, one-side: Ar–NO_2_ (1.0 mmol), FP4 9.45 wt% Pd loading MeOH : H_2_O (1 : 1, 5.0 mL); one-side: granular Zn (4.0 mmol), HCl 4 M (4.0 mL).

The difference in selectivity between methods A and B can be directly attributed to the set-up reaction in the two methods. The fact that the reducing agents in the two methods A and B were different, as well as the difference in the reduction potential of different functional groups, has led to high selectivity observed for the reduction of compounds. These factors have caused that in the simultaneous presence of two reducible functional groups, the reduction of one group was done selectively. In method B, where the reduction takes place in a sealed system in the presence of H_2_ gas, the conditions were also provided for the reduction of functional groups such as alkene, aryl halide, nitrile and oxime. For h and i compounds, since the stoichiometric amount of the reducing agent (H_2_ gas) was not used, a mixture of products h_1_, h_2_ and h_3_ as well as i_1_, i_2_, and i_3_ was obtained for the method B. In method A, due to the use of weaker ammonium formate reducing agent (under atmospheric pressure), it provides milder conditions for the reduction. The results of this study show that the filtration method can be used for the selective reduction of the aryl nitro in the simultaneous presence of oxime, hydroxyl, alkene, nitrile, and halide functional groups. However, in method B (under the sealed conditions under H_2_ gas), alkene and aryl halide groups can also be reduced. Another important factor that should be considered was the simultaneous presence of two reducible functional groups on the benzene ring (bi-functional substrates). Due to the difference in the reduction potential of different functional groups, simultaneous reduction of two different functional groups was done competitively. By reduction of one functional group on the substrate, it becomes an electron donor group, which can reduce the reactivity of the second functional group relative to the reducing agent.

### Control experiments

In order to ensure that the catalytic activity of the filter paper is unique, the catalytic activity of different materials used in the synthetic path of the catalytic filter paper was evaluated in the reduction of nitrobenzene to aniline. Table 7 shows the effect of the catalytic activity of Pd/C, FP@Si@C, FP@Si, FP, and AC as individual catalysts for both set-ups of filtration and sealed systems in the presence of ammonium formate and *ex situ* H_2_ generated respectively. Under identical conditions (the same as FP@Si@Pd/C filter paper), Pd/C nanoparticles produced only 65% and 70% conversion for methods A and B, respectively (Table 7, entry 1). In addition, the selectivity was significantly reduced, which was greater for method B. No significant efficiency was observed for FP@Si@C, FP@Si, FP, and AC (Table 7, entries 2–5). The results well demonstrate the unique catalytic activity of FP@Si@Pd/C in the presence of ammonium formate and H_2_ reducing agents as well as the vital role of Pd centers in the reduction of nitro compounds. Due to the lack of the catalytic activity for FP@Si@C, it was proved that Pd centers act as the catalytically active centers.

**Table 7:** Control experiments in the presence of Pd/C (10% wt), FP@Si@C, FP@Si, and cellulose filter paper as the catalyst for the reduction of nitrobenzene to aniline^a^

Entry	Catalyst	Method A			Method B		
		*t*. (min)	*C*. (%)	*S*. (%)	*t*. (min)	*C*. (%)	*S*. (%)
1^b^	Pd/C (10% wt)	190	65	80	8	70	65
2^c^	FP@Si@C	190/4	7	—	8	19	—
3^c^	FP@Si	190/4	5	—	8	12	—
4^c^	Cellulose filter paper	190/4	N.R.	—	8	N.R.	—
5^b^	Activated carbon	190	N.R.	—	8	N.R.	—

a General reaction conditions: nitrobenzene (1.0 mmol), MeOH : H_2_O (1 : 1, 5.0 mL), R.T.

b The reaction was performed heterogeneously by addition of 100 mg of the catalyst and stirred at room temperature. In order to evaluate the experiments under the same conditions, reactions were performed under a nitrogen atmosphere.

c The reactions using filter papers (entries 2–4) were performed on a water bath with 1.0 bar N_2_ inlet pressure. Other reactions were performed in a 10 mL round bottom balloon stirred with a magnetic stirrer bar under a N_2_ atmosphere. The loading of Pd (mmol/95 cm^2^) was about 1.0 ± 0.3.

Also, the results showed superior activity to reduce nitroarenes to aryl amines to the Pd-modified cellulose powder. Pd-supported perfluorohexyl modified cellulose catalyzed the reduction of nitrobenzene to *N*-phenylhydroxylamine (not to aryl amine) in the presence of NaBH_4_.^55^ In another report, hydrogenation of nitroarenes to aryl amines was catalyzed by cellulose supported Pd NPs for 5 h in the presence of a H_2_ atmosphere,^56^ in which the reduction efficiency decreased from 99 to 90% after 5 consecutive runs. In these non-magnetic heterogeneous systems, the catalyst recovery needs centrifugation or tedious filtration that causes the loss of the catalyst in each run. Also, contamination of NPs needs tedious and consecutive washing that leads to loss of catalyst mass.

Next, the effect of how to set up the reaction was studied. For this purpose, in the first set up, (1) the filter paper was cut into very small pieces and then used as a heterogeneous catalyst in the reaction mixture, and (2) in the second method, one filter paper was cut into pieces and suspended in the reaction mixture.

The results are summarized in Table 8. The set-ups are also applied for both ammonium formate and H_2_ reductants (Table 8). Considering the listed advantages for the filtration method, both methods created less efficiency than the filtration method. The results showed that the most effective setup to take advantage of the catalytic properties of the filter paper was its set-up as a filter paper on a glass funnel and the successive passage (filtration) of the reaction mixture.

**Table 8:** Examination of two other setups over the efficiency of nitrobenzene to aniline^a^

Set-up	Reductant					
	Ammonium formate			H_2_ (g)		
	*t*. (min)	*C*. (%)	*S*. (%)	*t*. (min)	*C*. (%)	*S*. (%)
Set up 1	190/4	75	98	8	80	95
Set up 2	190/4	45	95	8	60	92

a Reaction conditions: Reduction of nitrobenzene to aniline was performed according to the reported procedure presented in Tables 4 and 5, except the setup of the reaction (*i.e.* how to use the catalytic filter paper 4 in the reaction). Setup 1: the catalytic filter paper with 5.5 cm diameter was cut into small 1 cm-square pieces and added to the reaction mixture like a heterogeneous catalyst. Setup 2: catalytic filter paper with 5.5 cm diameter was cut into 8 conical pieces with a size of a 2.75 cm trim and held suspended by a clamp inside the reaction mixture.

It was also shown that in terms of how to set up the reaction, there was a significant difference between the cut paper and adding it to the reaction mixture or suspending it, and the efficiency for setup 1 was much better than that for setup 2 for both reductants (methods A and B). In addition, the setup could be upgraded for continuous flow reaction systems than the present batch reaction.

### Studies of reusability and stability

Due to the claim of portable application of the filter paper, it should be used many times. For this purpose, in the nitro benzene reduction to aniline, the catalytic filter paper was recovered and reused 4 consecutive times for both filtration and sealed system methods. As shown in Table 9, the efficiency loss in each cycle was very small and after 4 consecutive times, only 4 and 6% loss was observed for methods A and B, respectively. A noteworthy point was the lack of selectivity drop during successive cycles, which is of great importance in reduction reactions. However, the rate of efficiency declined during successive cycles that could be attributed to the reduction of the filter paper quality and the reduction in swellability (according to the results of leaching and swellability of the filter paper that will be discussed in the next section).

**Table 9:** Reusability evaluation of the catalytic FP@Si@Pd/C over the reduction of nitrobenzene to aniline using method A as well as method B^a^

Entry	Method A			Method B		
	*t*. (min)/NOF^b^	*C*. (%)	*S*. (%)	*t*. (min)/NOF^b^	*C*. (%)	*S*. (%)
1^th^ cycle	190/4	98	99	8	96	98
2^th^ cycle	190/4	96	99	8	94	98
3^th^ cycle	190/4	96	99	8	93	98
4^th^ cycle	190/4	95	99	8	90	98

a In all experiments only one filter paper was used.

b Number of filtration.

**Fig. 6:**
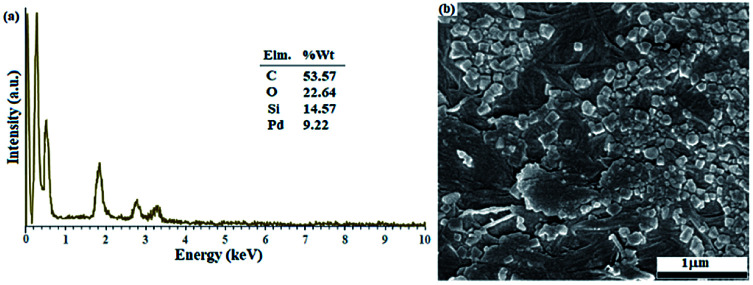
(a) EDX analysis and (b) FESEM analysis of FP@Si@Pd/C after 4 consecutive recycle tests over the reduction of nitro benzene to aniline (in method A).

The recovered filter paper was studied by EDX and FESEM analyses, to evaluate its structure and morphology respectively. As shown in Fig. 6, the filter paper not only retains the percentage composition of its elements, but also has exactly the same morphology as the freshly prepared paper. The filter paper owes this preservation of structure and properties to the successful silylation and immobilization of Pd/C on the cellulose fibers.

In order to study the catalytic activity of Pd centers on the surface of the filter paper as a heterogeneous catalyst, the Pd centers were poisoned with an excess amount of Hg(0).^57^ For this purpose, the catalytic filter paper in ethanolic solution of metallic mercury (0) (240 mmol) was shaken in a Petri dish for 2 h and then used in the nitrobenzene reduction to aniline in the presence of ammonium formate after complete drying. The results did not show any catalytic activity for 190 min (four consecutive filtrations) on the filter paper. Therefore, the results showed that the catalytic filter paper acts heterogeneously and the reactions take place specifically on the Pd catalytic centers. Also, due to the complete stop of the reaction, no Pd leaching took place on the filter paper.

**Scheme 4:**
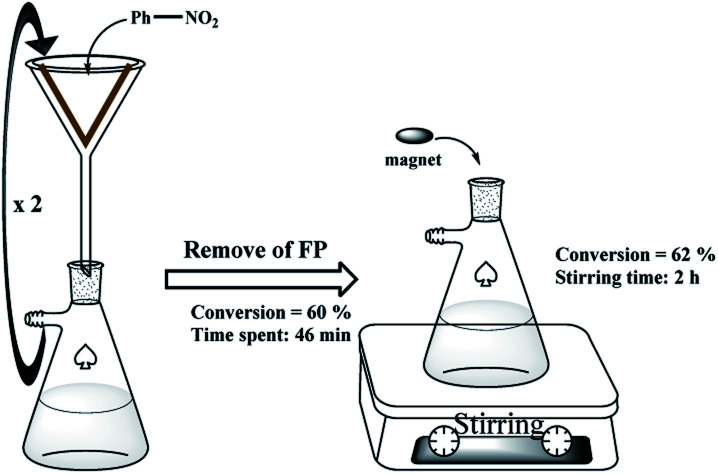
Hot filtration test on the FP 4.

The hot filtration test also confirmed the previous results and the lack of metal leaching from the filter paper. As shown in Scheme 4, in the nitrobenzene reduction by method A, the catalytic filter paper was removed after 2 consecutive filtrations, and then the reaction was stirred in the absence of the filter paper for 2 h with a magnetic stirrer. The results didn't show any reaction progress after this time which confirms lack of any metals into the reaction mixture.

**Fig. 7:**
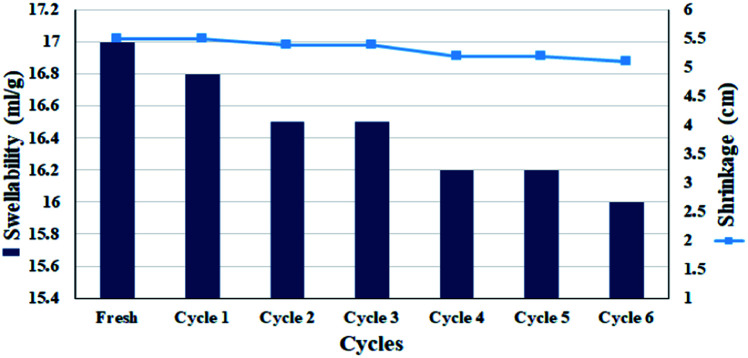
Swellability and shrinkage measurements of FP@Si@Pd/C in MeOH : H_2_O (1 : 1, v/v) for 6 consecutive drying–wetting cycles.

Due to the fact that the filter paper must be used several times, continuous wetting–drying affects the properties of the filter paper, especially its swelling rate.^58^ For this purpose, the swelling rate of the filter paper during successive cycles in MeOH : H_2_O (1 : 1) was studied. Chemical modifications on cellulosic fibrous have been shown to affect their % swelling.^59,60^ However, no significant reduction in swelling amount was observed in the filter paper for 6 consecutive wetting–drying cycles. This can be due to the presence of silica groups in the cellulose structure, which can act as a cross-linker between cellulose fibers, and also the presence of activated carbon with high water adsorption capability.^54,61^ Assuming that the CPTES group was attached to cellulose only through one of the methoxy groups, the other two groups have the potential to interact and subsequently crosslink between the cellulose fibers. This was also one of the reasons for the high swelling rate observed for solvents after silylation (Fig. 7). Therefore, this slight decrease in swelling could also be due to a change in the amount of crosslinking between the chains due to a change in the fibrous structure of the polymer during successive wetting–drying. Another factor was the closure of larger pores during re-wetting, which are unable to reopen and can cause surface tension forces.^58^ The results were completely consistent with the previously published reports on the swellability of cellulose paper after drying.^59^ However, the slight decrease in leaching has also been responsible for the slight decrease in nitro reduction efficiency. Re-measuring the diameter of the filter paper showed no significant shrinkage (diameter reduction of 1 mm) and the diameter of the filter paper after 5 consecutive wetting–drying cycles was 5.3 cm.

In order to ensure the absence of any metal leaching from the surface of the filter paper, its leaching was studied in various acidic, alkaline, and oxidative environments. As shown in Table 10, NaOCl, HNO_3_ (1 N), HCl (1 N), NaOH (1 N), and H_2_O_2_ 37% solutions were filtered through the FP@Si@Pd/C, and then both filter papers and the filtrate were studied by EDX and ICP analyses, respectively. The filter paper was completely stable against oxidative and alkaline environments, so that no leaching of Pd nor Si metals was observed in the filtrate. On the other hand, the EDX results obtained from the filter paper after exposure to these media also confirmed the composition of the elemental percentage of the filter paper the same as the freshly prepared filter paper. In acidic environments, the filter paper was also relatively stable. However, some leaching was observed in the presence of HNO_3_ and HCl, which was higher for HNO_3_. The results revealed that FP@Si@Pd/C filter paper could be used as a promising catalyst in all acidic, alkaline, and oxidative environments.

**Table 10:** Elemental composition study of FP@Si@Pd/C filter paper against acidic, basic, and oxidative reagents by ICP (from residue of filtrate) as well as EDX (mean of 5 points) analyses^a^

Entry	Sample	Reagent	EDX or ICP analysis (% wt of element)			
			C	Pd	Si	O
1	Residue (ICP analysis)	NaOCl	—	0	0	—
2		HNO_3_ (1 N)	—	3.88	3.45	—
3		HCl (1 N)	—	2.68	1.89	—
4		NaOH (1 N)	—	0	0	—
5		H_2_O_2_ 37%	—	0	0	—
6	FP@Si@Pd/C (EDX analysis)	NaOCl	53.52	9.31	14.54	22.63
7		HNO_3_ (1 N)	59.57	3.75	7.18	28.50
8		HCl (1 N)	54.73	7.27	10.90	24.10
9		NaOH (1 N)	53.50	9.27	14.62	22.61
10		H_2_O_2_ 37%	53.71	9.22	14.39	22.68

a For each reagent, 5 mL was used and the analyses were performed after 10 consecutive filtrations at room temperature.

In order to ensure the effect of the presence of Pd/C as well as silica groups in cellulose fibers on the reduction of shrinkage and swelling after successive wetting–drying, in a separate analysis, plain and silylated filter papers were also subjected to successive wetting and drying in EtOH : H_2_O solvent and their swelling rate was measured at each stage.

In accordance with the published articles, the plain filter paper undergoes a lot of shrinkage at each stage, and at the end of successive wetting–drying cycles (5 consecutive cycles) the paper undergoes about 7 mm decrease in diameter, and the swelling rate at 13.3 mL g^−1^ reaches 8.6 mL g^−1^.

But for the silylated filter paper, the amount of shrinkage was equal to 2 mm and the amount of swelling reduced to 12, which was insignificant. Surface silylation stops the shrinkage and subsequently preserves swelling by creating a possible crosslinking (ESI, Fig. S7†).

Finally, the effect of pH on metal leaching from the filter paper was studied. For this purpose, using HCl and NaOH solutions, the filter paper leaching was measured at a pH in the range of 1–14. The results were shown in terms of Pd% wt leached from the filter paper (Figure S8†). In each reaction, 5 mL of each prepared pH was filtered 5 times and then the reaction mixture was studied by atomic absorption analysis for Pd leaching. The maximum amount of Pd leaching at pHs 1,2 was equal to 9.45% wt Pd (approximately equal to the total amount of Pd loaded on the filter paper). The leaching amount was decreased at pH 2 and 3 equal to 9.4 and 8.8% wt Pd. At pH 4 to 14, no leaching was observed, indicating the relative stability of the filter paper in acidic media and high stability in alkaline environments. According to the results, the catalytic filter paper prepared in this study can be used in mild and acidic environments with confidence.

### Mechanism studies

According to the previously reported mechanisms of nitro reduction on a heterogeneous system,^5,12,20,62^ a possible mechanism for the reduction of nitroarenes was presented by both methods A and B in the presence of ammonium formate and H_2_ gas, respectively. In the presence of a catalytic amount of Pd/C, ammonium formate undergoes decomposition to CO_2_, NH_3_ and H_2_.^20,63^ This type of hydrogenation reaction was typically performed in a solvent by stirring a heterogeneous mixture until the reaction was complete. The formate ion could be adsorbed on the Pd/C surface in the first step of the mechanism (Pd(HCOO^−^)_ad_).^64,65^ In the filtration method, protons are first adsorbed on the Pd centers in the presence of the formate group at Pd/C sites (Pd (H^−^) intermediate).

**Scheme 5:**
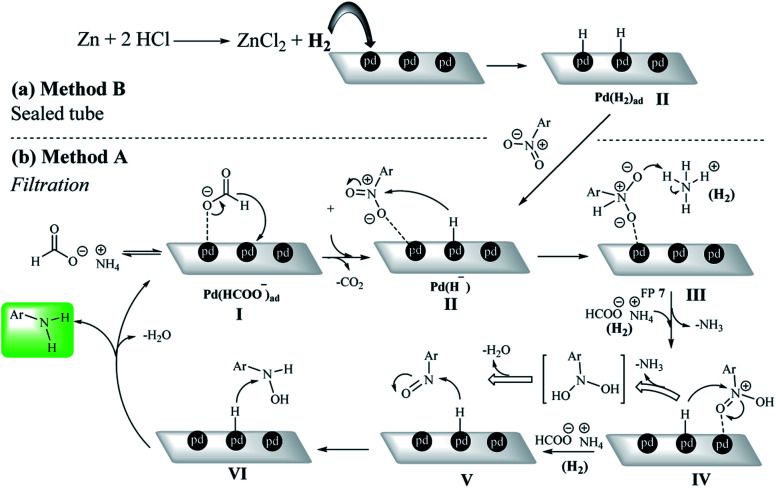
Two plausible reaction mechanisms for the nitrobenzene reduction by (a) sealed (method B) and (b) filtration methods (method A).

In method B, the Pd(H^−^) intermediate was created by *ex situ*-H_2_ generated by the reaction of Zn with HCl (Scheme 5). Due to the strong interaction of Pd with O, nitro groups are adsorbed on the catalyst surface by oxygen (intermediate III). Then, in the presence of ammonium formate (method A) or hydrogen gas (method B), intermediate IV is formed.^66–69^ Studies from the control experiments have shown that the reaction definitely takes place on the Pd centers and also the reductant was a mandatory reagent for the nitro reduction (Scheme 5).

## Conclusion

Transfer hydrogenation of nitroarenes was performed on a portable catalytic filter paper in the presence of ammonium formate as well as H_2_ gas as two individual protocols. In the presence of ammonium formate, the reaction was performed by several consecutive filtrations of the reaction mixture, for the first time. In the presence of *ex situ*-H_2_ generated gas in a sealed two-chamber H-tube system, the halogenated nitroarenes were reduced entirely. Hydrogenation of olefins, and nitrile reduction to amine could also be performed by the present protocol. High selectivity and efficiency were obtained for a wide variety of nitroarenes in the presence of both ammonium formate and H_2_ gas. In the filtration method, the nitro group can be selectively reduced to amine without interference of CN, OH, olefin, halogen, and oxime functional groups. The filter paper under a H_2_ atmosphere had a selectivity of X = NO_2_> => CN > N = –OH. The results showed that the most effective setup to take advantage of the catalytic properties of the filter paper was its set-up as a filter paper on a glass funnel and the successive passage (filtration) of the reaction mixture. The results from leaching, swellability, pH tests, and recyclability studies on the filter paper confirm its stability in order to be used several times. Another advantage of the catalytic filter paper as a portable catalyst was its possibility to be used in industry even by non-experts. In method B, it was shown that catalytic filter paper can also be used as a heterogeneous catalyst and its presence in the reaction mixture causes it to be catalyzed. The results revealed that FP@Si@Pd/C filter paper could be used as a promising catalyst in all acidic, alkaline, and oxidative environments.

## Experimental section

### Instrumentation

A circle folded Whatman cellulose filter paper (Whatman 1201-320) grade 1V, with 0.2 mm thickness, 32 cm diameter, 8–10 micron, 150 s/100 mL speed (Herzberg) with a basic weight of 120 g m^−2^, was purchased from Sigma for preparation of the catalytic filter paper. Functionalized (acid treated) activated carbon (mesh size −100) was purchased from LobalCheme (Mumbai, India). An Orbital Shaker, 10 mm Orbit from BTLabSystems, was used for shaking of the samples. The reaction setup was equipped with a low pressure N_2_-inlet (0–1 bar) V-121K091 R-21 regulator (inlet connection W2432), to control the filtration rate (contact time duration). FTIR as well as ATR (in the case of filter papers) spectra were recorded on a JASCO FT/IR 4600 spectrophotometer. The NMR (^1^H and ^13^C) spectra were recorded using a Bruker AVANCE III 300 MHz spectrometer in deuterated solvents including CDCl_3_ and DMSO-*d*_*6*_. TMS was used as an internal standard. ICP analyses were performed on a NexION 2000B ICP mass spectrometer. Crystal structure of the samples was studied by X-ray diffraction (XRD) patterns of the samples on a Rigaku Smart-Lab X-ray diffractometer with Cu Kα (*λ* = 1.5418 nm) radiation. Morphology of the samples was studied by the field emission scanning electron microscopy (FESEM) technique using Tescan MIRA3 apparatus. Elemental analysis of the samples was performed using EDX spectroscopy on a JEOL 7600F field emission scanning electron microscope, equipped with a spectrometer of energy dispersion of X-ray from Oxford Instruments. Experimental design for the statistical studies over the effective parameters on the nitro reduction as well as subsequent regression analyses of the experimental data was performed using Design-Expert statistical software version 11, Stat-Ease Inc., Minneapolis, MN, USA. Gas chromatography analyses were performed on a YL 6100 gas chromatograph system (GC) with a CBP5 column (Shimadzu 30 m × 0.32 mm × 0.25 mm). The conversion and selectivity of the products were determined using GC software (Autochro-3000).

Selectivity of the products (Table 6) was measured by gas chromatography. For each experiment, the resulting mixture (0.2 μL) was injected to GC instrument and then the selectivity of the products was calculated by the following equation:^70^
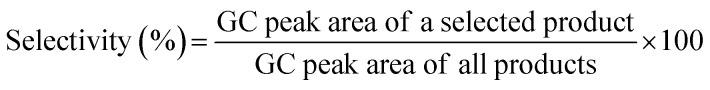


### Silylation of cellulose filter paper (FP@Si)

For the silylation of cellulose FP, two protocols could be used:

(1) Silylation of cellulose filter paper was inspired by the work of Chantereau *et al.*^46^ in water. The plain filter paper (Whatman 1201-320, grade 1V, with 0.2 mm thickness, 32 cm diameter, 8–10 micron, 150 s/100 mL speed (Herzberg) with a basic weight of 120 g m^−2^) was soaked in a basicified (with NH_3_ solution, pH = 9.0) aqueous solution of (3-chloropropyl)triethoxysilane (340 mM). The setup was shaken for 24 h at room temperature to complete adsorption/deposition of CPTES on the FP. Then, the paper was freeze-dried for 24 h and stored in a desiccator with P_2_O_5_ before use.

(2) In the second protocol, a simple chemical vapor deposition was performed according to a previously reported protocol with some modifications.^47^ This method was used to expose CFP to the CPTES vapor in the absence of any catalyst at an elevated temperature. 25 mL of CPTES was added to a closed chamber bearing an open vessel holding the CPTES. Then, a plain CFP was placed on a porous filter paper and the temperature was adjusted to 215 °C. The test was repeated at three different time intervals of 2, 6, 8, and 12 h. The cellulose filter papers treated by vapor deposition were marked as SiCFPvap.

### Preparation of activated carbon-supported Pd

Carbon-supported palladium (Pd/C) was prepared by a previously reported liquid-phase reduction with slight modification.^52^ Briefly, in a round-bottom flask, functionalized carbon (0.4 g) was dispersed in 100 mL of deionized water. Then, the pH of the solution was adjusted to 11 using ammonia solution (28–30 wt%) to get a net negatively charged carbon surface. The reaction mixture was stirred for 2.0 h at room temperature. To prepare Pd/C particles with 10 wt% Pd loadings, 19% wt (NH_3_)_4_Pd(NO_3_)_2_ was added to the mixture, and the mixture was stirred for 24 h. Subsequently, aqueous reducing agent solutions (excess amounts) including formaldehyde (37 wt%, 3 mL) and NaBH_4(aq)_ (0.5 mmol in 5 mL, freshly prepared) were added, respectively at room temperature. The first reductant was added instantly, and the reaction mixture was stirred for 1 h at 80 °C. Then, NaBH_4_ solution was added dropwise for 5.0 min and the reaction mixture was stirred at room temperature for 1 h. The resulting Pd/C product was separated by centrifugation (20 000 rpm), and washed with deionized water several times, and then dried at 80 °C in a vacuum oven.

### Preparation of FP@Si@Pd/C catalytic filter paper *via* route A

Pd/C was immobilized on the silylated filter paper in one step. In a 10 cm diameter glassy Petri dish, Pd/C (200 mg) was dispersed to 15 mL of ethanolic ammonia solution. The presence of ammonia also helps to neutralize the acid produced during CPTES coupling to the hydroxyl groups in the cellulose fibers. Then, the silylated cellulose filter paper with 5.5 cm diameter was slowly inserted into the Petri dish. In order to immobilize the Pd/C into the filter paper, the Petri dish was shaken for 48 hours at room temperature. FP@Si@Pd/C catalytic filter paper was dried and stored in a vacuum desiccator containing P_2_O_5_ as a desiccant and dehydrating agent.

### Immobilization of activated carbon on the silylated FP (FP@Si@AC) and the preparation of FP@Si@Pd/C catalytic filter paper *via* route B

To immobilize AC on the silylated filter paper, 200 mg of AC was dispersed in 15 mL of ethanolic ammonia solution and added to a 10 cm diameter glassy Petri dish. Then, the silylated cellulose filter paper with 5.5 cm diameter was slowly inserted into the Petri dish. In order to immobilize the AC into the filter paper, the Petri dish was shaken for 48 hours at room temperature. FP@Si@C filter paper was dried at 45 °C in a vacuum oven. To coordinate Pd ions with 10 wt% Pd loadings into the FP@Si@C filter paper, 19% wt (NH_3_)_4_Pd(NO_3_)_2_ aqueous solution was added to the Petri dish, and then the FP@Si@C filter paper was inserted to the dish. The dish was shaken for 24 h at room temperature. Subsequently, aqueous reducing agent solutions (excess amounts) including formaldehyde (37 wt%, 3 mL) and NaBH_4(aq)_ (0.5 mmol in 5 mL, freshly prepared) were added, respectively at room temperature to the dish. The first reductant was added instantly, and the reaction mixture was shaken for 4 h. Then, NaBH_4_ solution was added dropwise for 5.0 min to the reaction mixture at room temperature and then shaken for 1 h. The mixture containing FP@Si@Pd/C catalytic filter paper was shaken for an additional 12 h at ambient temperature. The resulting FP@Si@Pd/C filter paper was separated and washed with deionized water and cool EtOH several times, and then dried at 45 °C in a vacuum oven.

### General procedure for nitro reduction catalyzed by FP@Si@Pd/C catalytic filter paper by the filtration protocol

As a typical procedure for the FP@Si@Pd/C-catalyzed nitro reduction, in a glassy vacuum Erlenmeyer flask (100 mL) Ar–NO_2_ (1.0 mmol) and ammonium formate (10.0 mmol) were dissolved in a solution of MeOH : H_2_O (1 : 1, 5.0 mL) at room temperature. The modified folded filter paper (circle with 5.5 cm diameter and 0.2 mm thickness), FP@Si@Pd/C, was placed on a glass funnel (as shown in Scheme 2), all on a vacuum Erlenmeyer flask. Also, the vacuum Erlenmeyer flask was equipped with a low pressure N_2_ inlet (1.0 bar, V-121K091 R-21 regulator (inlet connection W2432)) to control the filtration rate. The reaction mixture was poured on the filter paper at once, in one step, so that the entire surface of the filter paper was covered by the reaction mixture (the reaction mixture rose to near the edge of the filter paper). At each filtration, the reaction progress was monitored by TLC and GC. In each filtration cycle, a glass plate was placed (sealed) on the funnel to avoid solvent evaporation. After reaction completion, the filter paper was washed with 10 mL of hot absolute ethanol. Scheme 2 shows the general schematic of the setup for the reaction of the nitro reduction. The reaction time was accurately calculated using a stopwatch and only during the contact of the reactants with the filter paper (filtration time only). For each filtration, the time was calculated immediately after pouring the reactants on the filter paper and the time was stopped after the filtration was completed. The next cycle was repeated in the same way. Due to the constant pressure in Erlenmeyer, the filtration follows a constant mean time.

### General procedure for nitro reduction catalyzed by FP@Si@Pd/C catalytic filter paper by *ex situ* H_2_ production in a H-tube

In a COware, H-tube,^26^ FP@Si@Pd/C catalytic filter paper (FP4 9.45 wt% Pd loading) was placed in the bottom of the one-side of the H-tube as a U-shape. One-side of the H-tube containing the filter paper 4 was charged with Ar–NO_2_ (1.0 mmol) and MeOH : H_2_O (1 : 1, 5.0 mL); the other-side was charged with granular Zn (4.0 mmol) and HCl 4 M (4.0 mL). The mixture above the paper was stirred mechanically using a mechanical stirrer that passed through the plastic cap of the H-tube. The mixture was stirred under completely sealed conditions at room temperature. To monitor the reaction progress, in each stage, the tap between the two sides of the H-tube was closed, and the progress of the reaction was monitored by GC or TLC. Upon the reaction completion, the filter paper was removed using forceps, and washed with 10 mL of hot absolute ethanol, and then the resulting residue was added to the reaction mixture to accurately determine the reduction efficiency (due to possible contamination of the FP by reactant and/or product(s)) (Scheme 2). Then, the FP4 was stored and dried at 45 °C in a vacuum oven for the next use.

## Conflicts of interest

There are no conflicts to declare.

## Supplementary Material

RA-012-D2RA01151D-s001
