# Supplementary material for: Transfer hydrogenation of nitroarenes using cellulose filter paper-supported Pd/C by filtration as well as sealed methods
Source: RSC Adv. 2022 Apr 7;12(18):10933–49. doi: 10.1039/d2ra01151d (PMC8988863; doi:10.1039/d2ra01151d)
Supplement: RA-012-D2RA01151D-s001 [file RA-012-D2RA01151D-s001.pdf]

Electronic Supplementary Material (ESI) for RSC Advances.
This journal is © The Royal Society of Chemistry 2022

Transfer Hydrogenation of nitroarenes using cellulose filter paper-supported Pd/C by filtration as well as sealed methods

Dmitry Olegovich Bokov,[a] Mustafa Z. Mahmoud,[b,c] Gunawan Widjaja,[d,e] Wanich Suksatan,[f] Supat Chupradit,[g] Usama S. Altimari,[h] Hussein Ali Hussein,[i] Yasser Fakri Mustafa,[j] Milad kazemnejadi*[k]

[a.] Institute of Pharmacy, Sechenov First Moscow State Medical University, 8 Trubetskaya St., bldg. 2, Moscow, 119991, Russian Federation.
[b.] Department of Radiology and Medical Imaging, College of Applied Medical Sciences, Prince Sattam bin Abdulaziz University, Al-Kharj 11942, Saudi Arabia
[c.] Faculty of Health, University of Canberra, Canberra, ACT, Australia.
[d.] Postgraduate Study, Universitas Krisnadwipayana, Bekasi, Indonesia.
[e.] Faculty of Public Health, Universitas Indonesia, Depok, Indonesia.
[f.] Faculty of Nursing, HRH Princess Chulabhorn College of Medical Science, Chulabhorn Royal Academy, Bangkok, Thailand.
[g.] Department of Occupational Therapy, Faculty of Associated Medical Sciences, Chiang Mai University, Chiang Mai, 50200, Thailand.
[h.] Al-Nisour University College, Baghdad, Iraq.
[i.] Scientific Research Center, Al-Ayen University, Thi-Qar, Iraq.
[j.] Department of Pharmaceutical Chemistry, College of Pharmacy, University of Mosul, Mosul-41001, Iraq.
[k.] Department of Chemistry, College of sciences, Shiraz University, Shiraz 71946-84795 Iran.
Corresponding author: E-mail: miladkazemneja@yahoo.com (M. Kazemnejadi)

Swelling measurements of filter papers

There are two ways to calculate swelling of a film, including (i) calculation of the volume changes of a film before and after swelling [1] and (ii) calculation of weight changes in film in the solvents [2]. Using equation 1, swelling volume of the filter papers (in mL/g) was calculated [3]:

$$V_s = \frac{W_2 - W_1}{W_1} \times \frac{1}{d_s} \qquad \text{(Eq. 1)}$$

Where V_s, W_2, W_1 and ds are swelling amount of the filter paper (mL/g), weight of swollen network (gr), weight of dry filter paper (gr) and density of the solvent, respectively.

Practically, the dry filter paper was weighted to get W_1 and then immersed in the desired solvents for 2 h at room temperature to determine W_2. With having density of each solvent, the calculation of volume per grams becomes possible.

Other procedures

In order to benefit from the highest catalytic activity of the filter paper, in addition to the filtering protocol, two other protocols of (1) the cutted filter paper and using it in the reaction mixture (like a heterogeneous catalyst) and (2) suspending the filter paper pieces inside the reaction mixture [4] were also studied and compared.

(1) In the first set up, the filter paper was cut into several 1 cm square pieces and added to the reaction medium as a heterogeneous catalyst. The reaction was stirred in the presence of a magnet bar and its progression was studied by GC or TLC analyses. In this method, the filter papers were separated from the reaction medium by forceps in the end of the reaction and in order to achieve the maximum efficiency, the filter paper pieces were placed in a plate containing 15 ml of pure ethanol for 5 hours and the plate was shacked for 1 hour. The resulting solution was then added to the reaction mixture to determine the conversion percentage.

(2) In this case, the filter paper with a diameter of 5.5 cm in accordance with the method presented by Nishikata *et al.* [4] was kept inside the reaction mixture by a clamp, with the difference that in order to make a logical comparison with the other two methods (because Pd loading is depends on the surface area of the catalyst), a sheet of the catalytic filter paper **4**, divided into 8 equal cone-shaped parts with a rim size of 2.75 cm and held suspended by a clamp inside the reaction mixture. The reaction was stirred by a magnetic stirrer and the progress of the reaction was monitored by GC or TLC. As in set up 1, the filter paper pieces were shaken in 15 ml of pure ethanol to achieve the highest efficiency (to ensure that the filter paper contained no products) and the resulting solution was added to the reaction mixture.

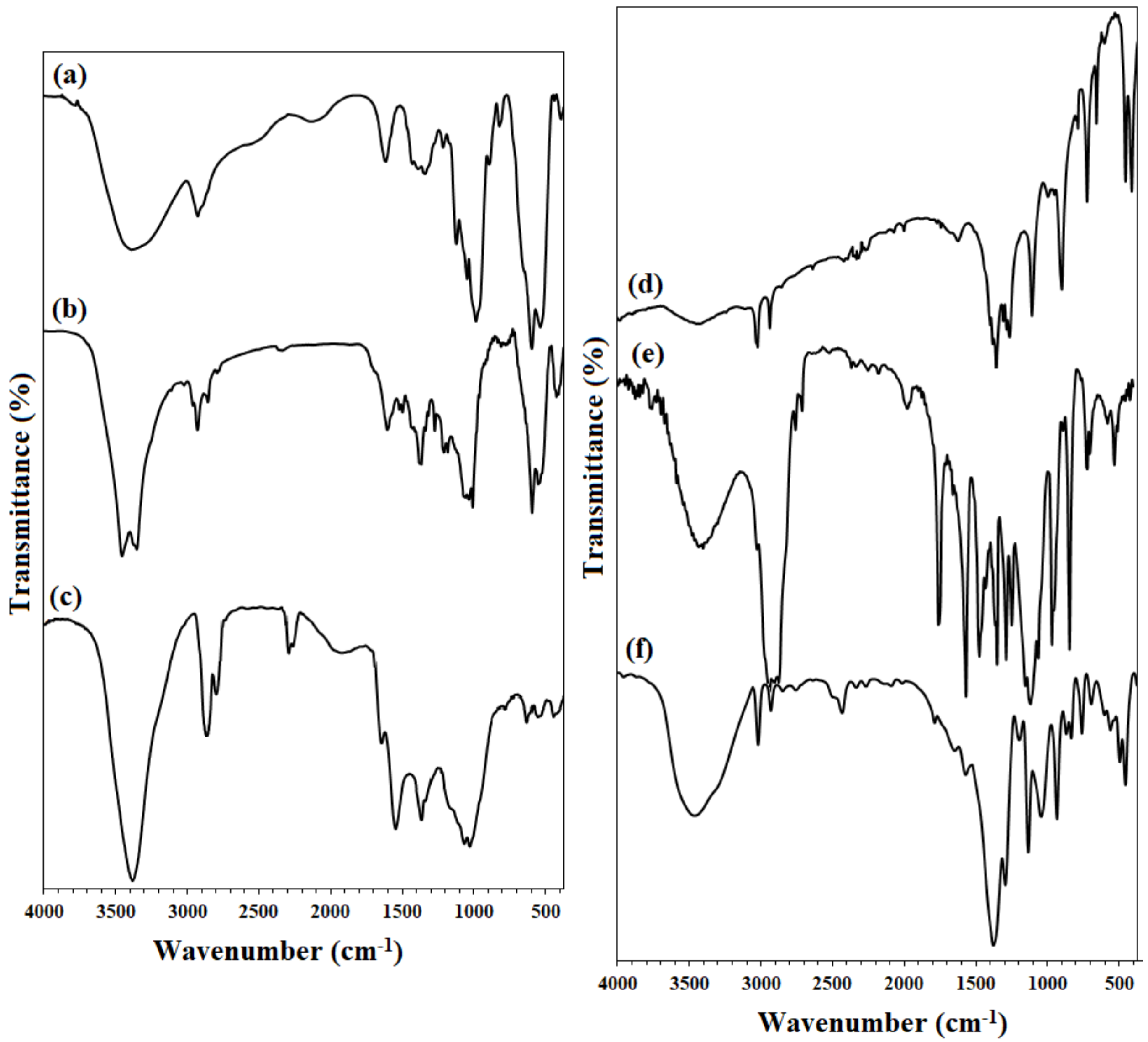

Fig. S1 FTIR spectra of (a) plain filter paper, (b) silylated filter paper (FP@Si), (c) activated carbon (AC), (d) Pd/C, (e) FP@Si@AC, and (f) FP@Si@Pd/C

The presence of only C, O elements in the filter paper indicates the purity and the absence of any impurities in the paper (Figure S2-a).

According to the results of EDX analysis, the presence of 11.36%wt Si in the silylated filter paper, not only confirms the success of the silylation process, but also the high amount of loading allows the optimal immobilization of the activated carbon (or Pd/C) with a high loading percentage. The presence of the Cl-related peaks at the 2.6 eV (Cl Kα) and 2.8 eV (Cl Kβ) binding energies were another confirmation of the successful immobilization of the CPTES on the filter paper (Figure S2-b).

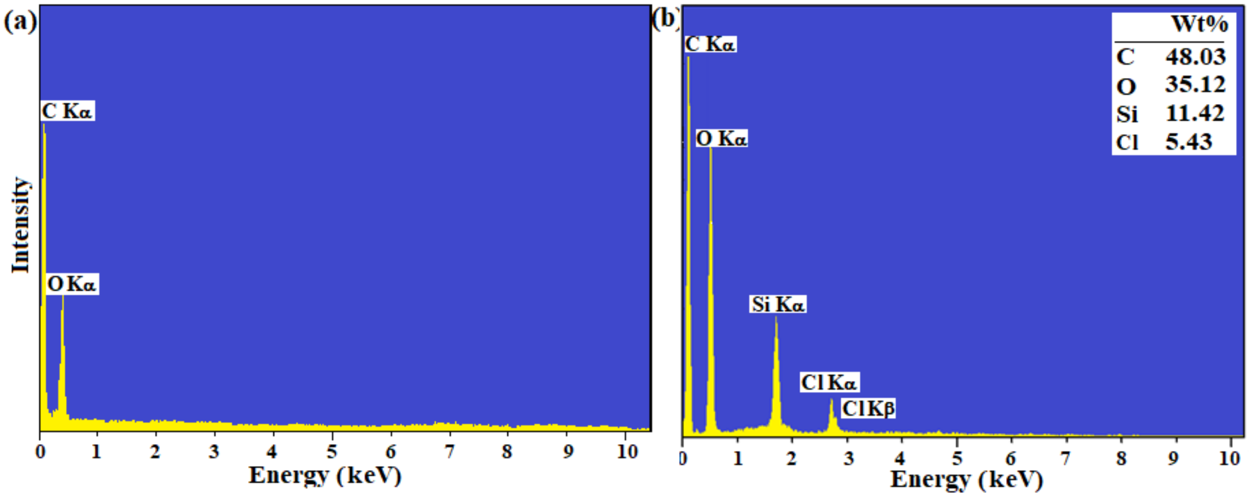

Fig. S2 EDX spectra of (a) plain and (b) silylated (FP@Si) cellulose filter paper. (The inset table represents the mean of five points for the elemental composition).

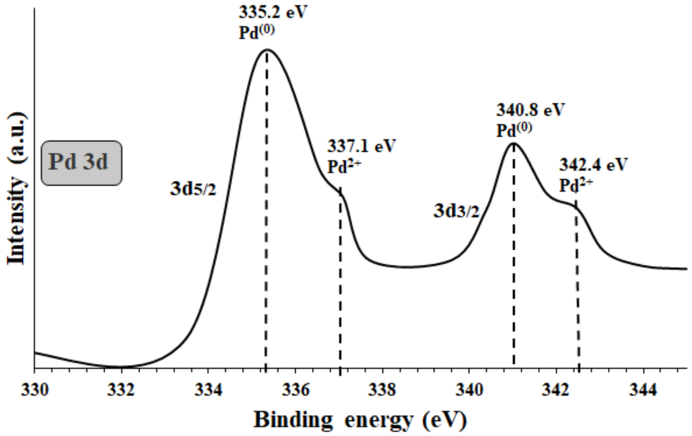

Fig. S3 High resolution XPS analysis (energy corrected, normalized) of Pd3d on the as-prepared Pd/C NPs

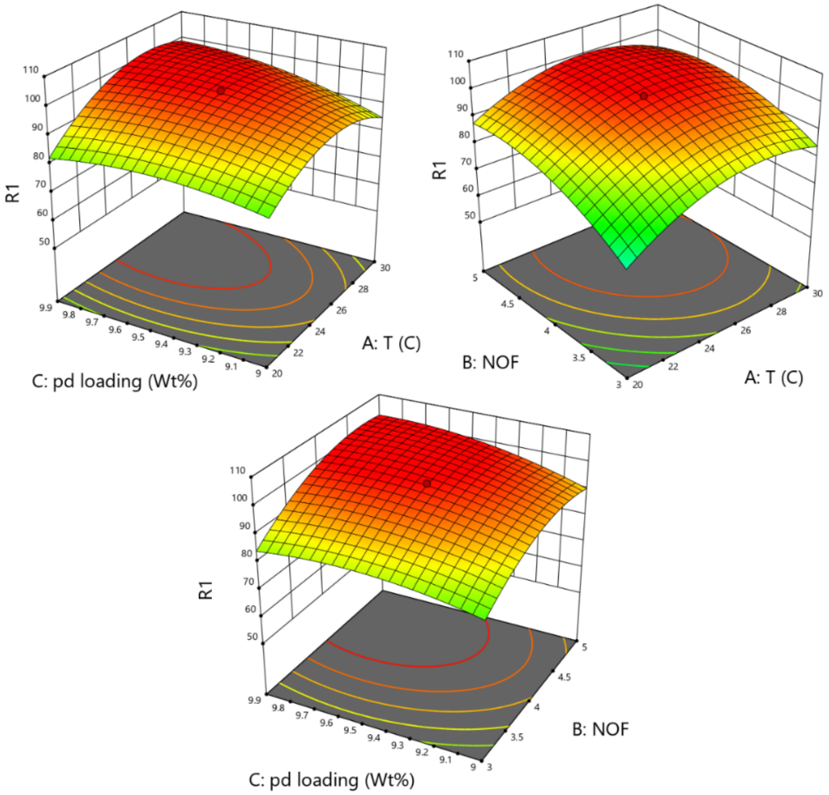

Fig. S4 The 3D central design curves for foundation of optimization ranges for maximum response in model nitrobenzene reduction to aniline (R1)

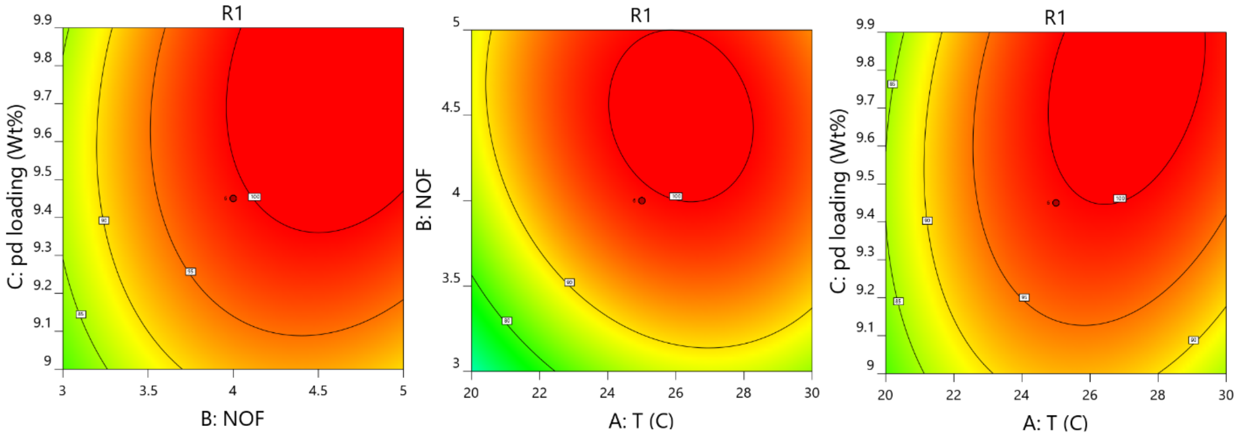

Fig. S5 The 2D central design curves for foundation of optimization ranges for maximum response in model nitrobenzene reduction to aniline (R1)

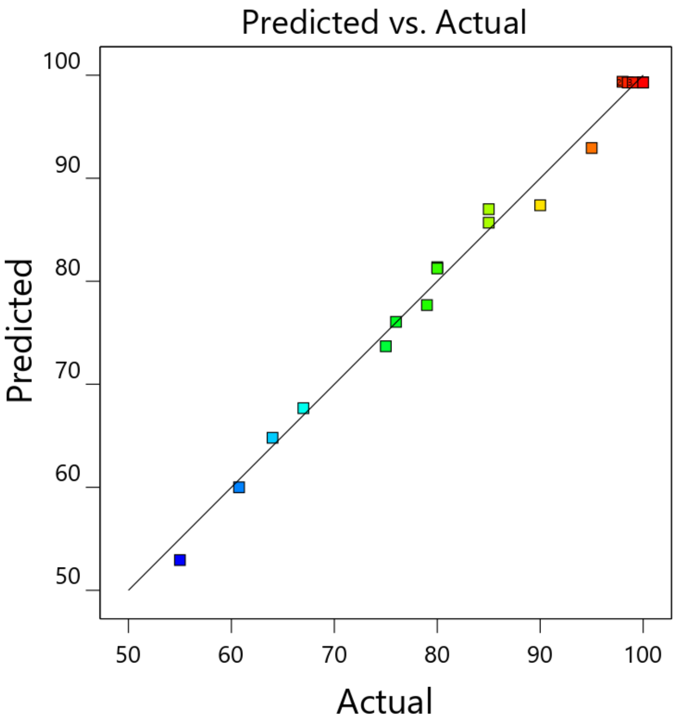

Fig. S6 Accuracy of the predicted model *vs*. actual values

Table S1. Sequential Model Sum of Squares [Type I]

Source	Sum of Squares	df	Mean Square	*F*-value	*p*-value	
Mean vs Total	1.422E+05	1	1.422E+05			
Block vs Mean	89.72	2	44.86			
Linear vs Block	1412.52	3	470.84	2.58	0.0948	
2FI vs Linear	150.38	3	50.13	0.2296	0.8738	
**Quadratic vs 2FI**	**2387.75**	**3**	**795.92**	**471.79**	**< 0.0001**	**Suggested**
Cubic vs Quadratic	4.81	4	1.20	0.5533	0.7098	Aliased
Residual	8.69	4	2.17			
Total	1.462E+05	20	7311.29			

Table S2. ANOVA for Quadratic model

Source	Sum of Squares	df	Mean Square	*F*-value	*p*-value	
Block	89.72	2	44.86			
**Model**	3950.64	9	438.96	260.20	< 0.0001	significant
A-T	430.56	1	430.56	255.22	< 0.0001	
B-NOF	749.39	1	749.39	444.21	< 0.0001	
C-pd loading	232.56	1	232.56	137.85	< 0.0001	
AB	36.13	1	36.13	21.41	0.0017	
AC	78.13	1	78.13	46.31	0.0001	
BC	36.13	1	36.13	21.41	0.0017	
A^2	1941.75	1	1941.75	1151.00	< 0.0001	
B^2	983.36	1	983.36	582.90	< 0.0001	
C^2	293.13	1	293.13	173.75	< 0.0001	

**Residual**	13.50	8	1.69			
Lack of Fit	11.37	5	2.27	3.21	0.1829	not significant
Pure Error	2.13	3	0.7083			
**Cor Total**	4053.86	19				

Factor coding is **Coded**.
Sum of squares is **Type III - Partial**
The **Model F-value** of 260.20 implies the model is significant. There is only a 0.01% chance that an F-value this large could occur due to noise.
P-values less than 0.0500 indicate model terms are significant. In this case A, B, C, AB, AC, BC, A^2, B^2, C^2 are significant model terms. Values greater than 0.1000 indicate the model terms are not significant. If there are many insignificant model terms (not counting those required to support hierarchy), model reduction may improve your model.
The **Lack of Fit F-value** of 3.21 implies the Lack of Fit is not significant relative to the pure error. There is a 18.29% chance that a Lack of Fit F-value this large could occur due to noise. Non-significant lack of fit is good -- we want the model to fit.

Table S3. Fit Statistics

**Std. Dev.**	1.30		**R^2**	0.9966
**Mean**	84.31		**Adjusted R^2**	0.9928
**C.V. %**	1.54		**Predicted R^2**	0.9719
			**Adeq Precision**	46.0738

The **Predicted R^2** of 0.9719 is in reasonable agreement with the **Adjusted R^2** of 0.9928; i.e. the difference is less than 0.2.
Adeq Precision measures the signal to noise ratio. A ratio greater than 4 is desirable. Your ratio of 46.074 indicates an adequate signal. This model can be used to navigate the design space.

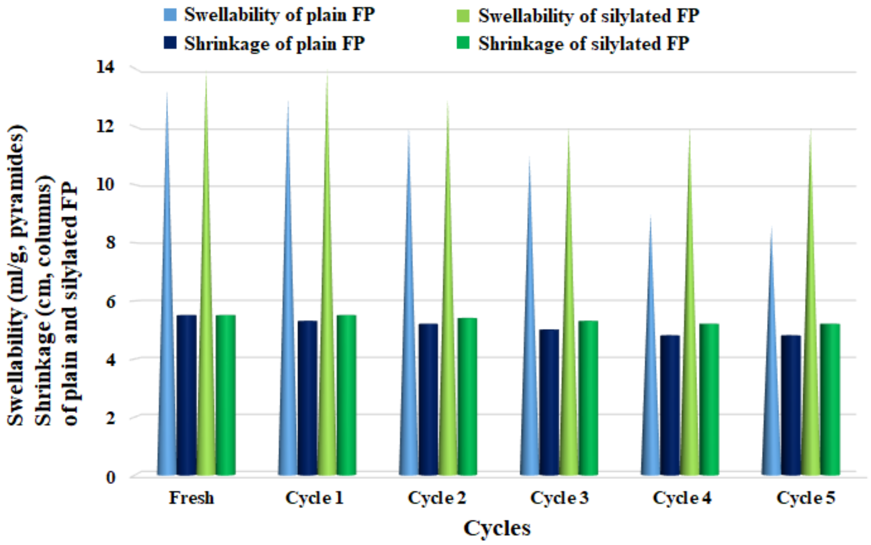

Fig. S7 Swellability and shrinkage of the plain (unmodified) and silylated filter paper in EtOH: H_2O solvent for 5 consecutive wetting-drying cycles

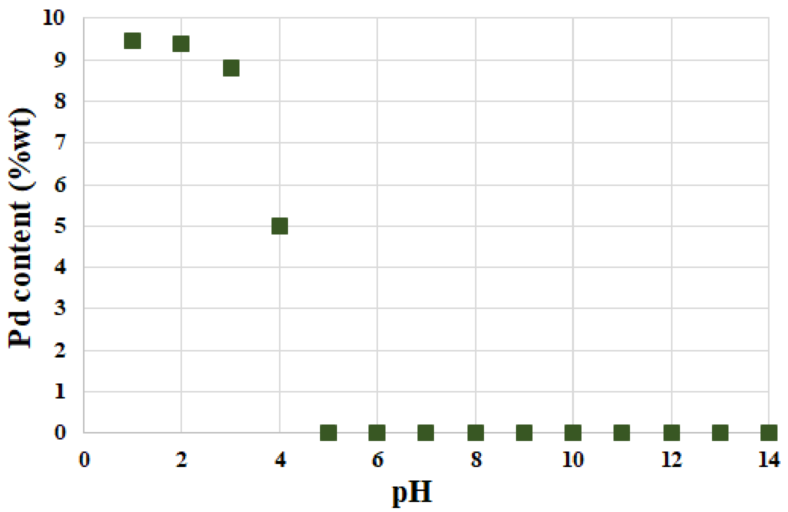

Fig. S8 Influence of pH over Pd leaching of FP@Si@Pd/C

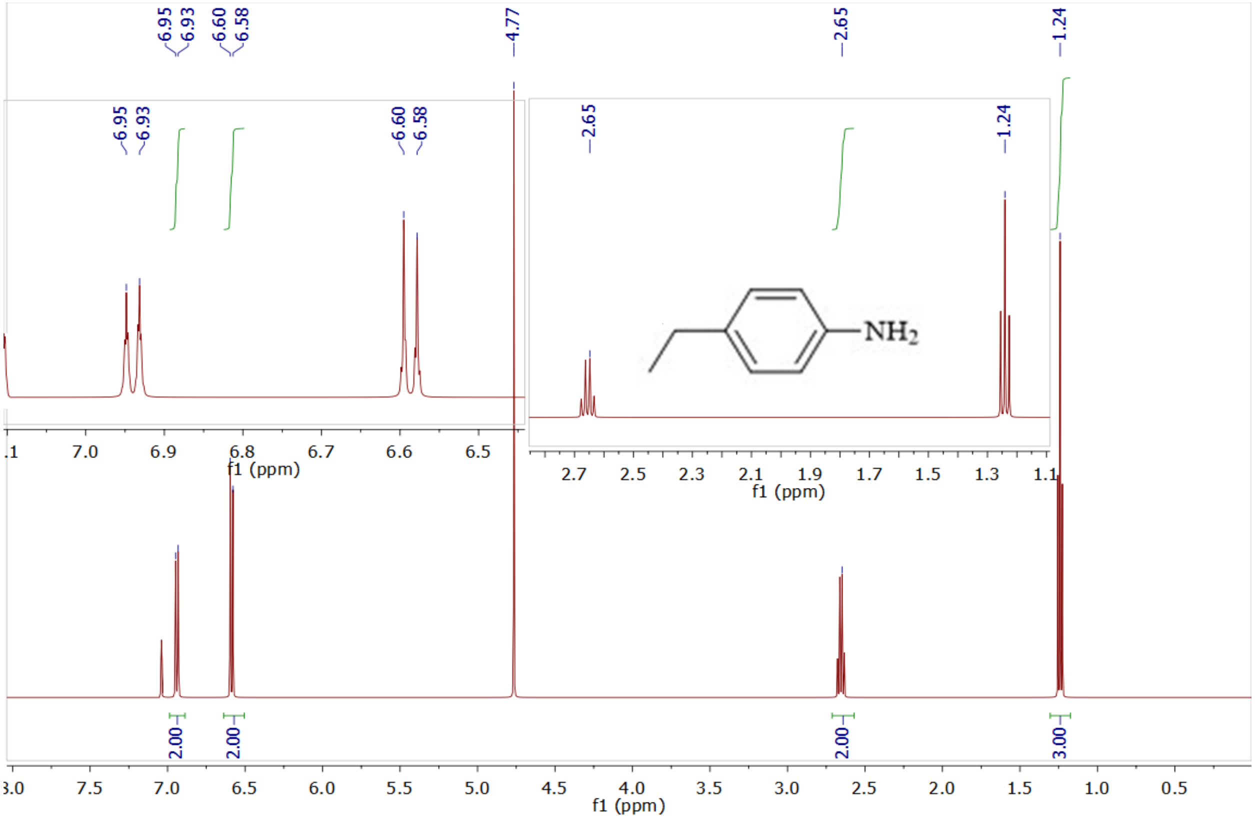

Fig. S9 ^{1}HNMR spectrum of d_1 (300 MHz, $CDCl_3$) (Table 6)

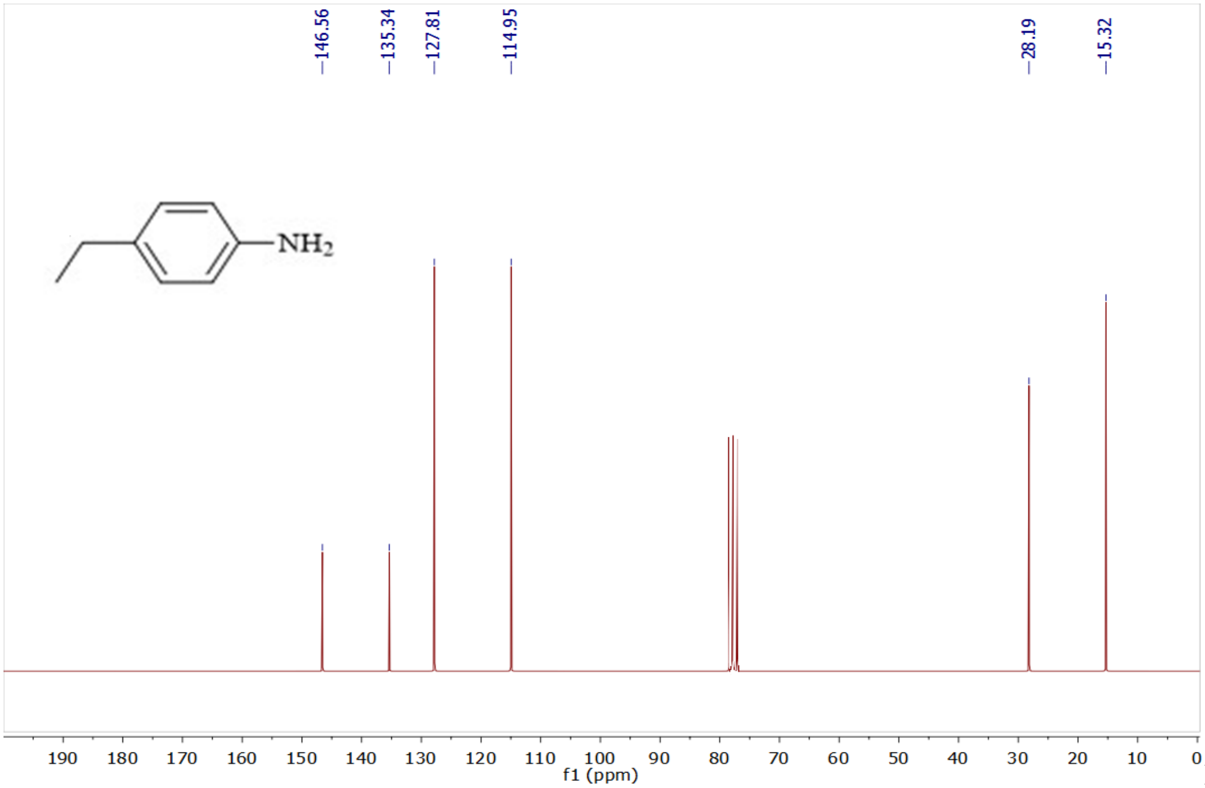

Fig. S10 ^{13}CNMR spectrum of d_1 (75 MHz, $CDCl_3$) (Table 6)

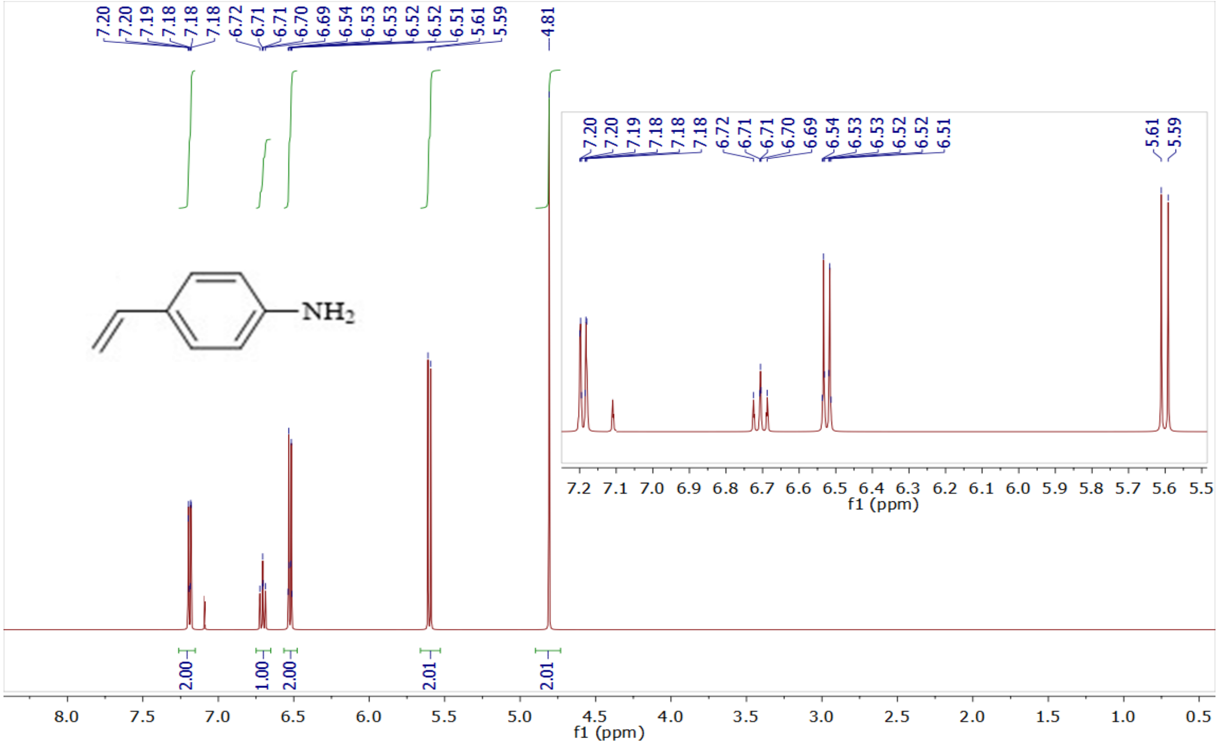

Fig. S11 ^{1}HNMR spectrum of d_2 (300 MHz, $CDCl_3$) (Table 6)

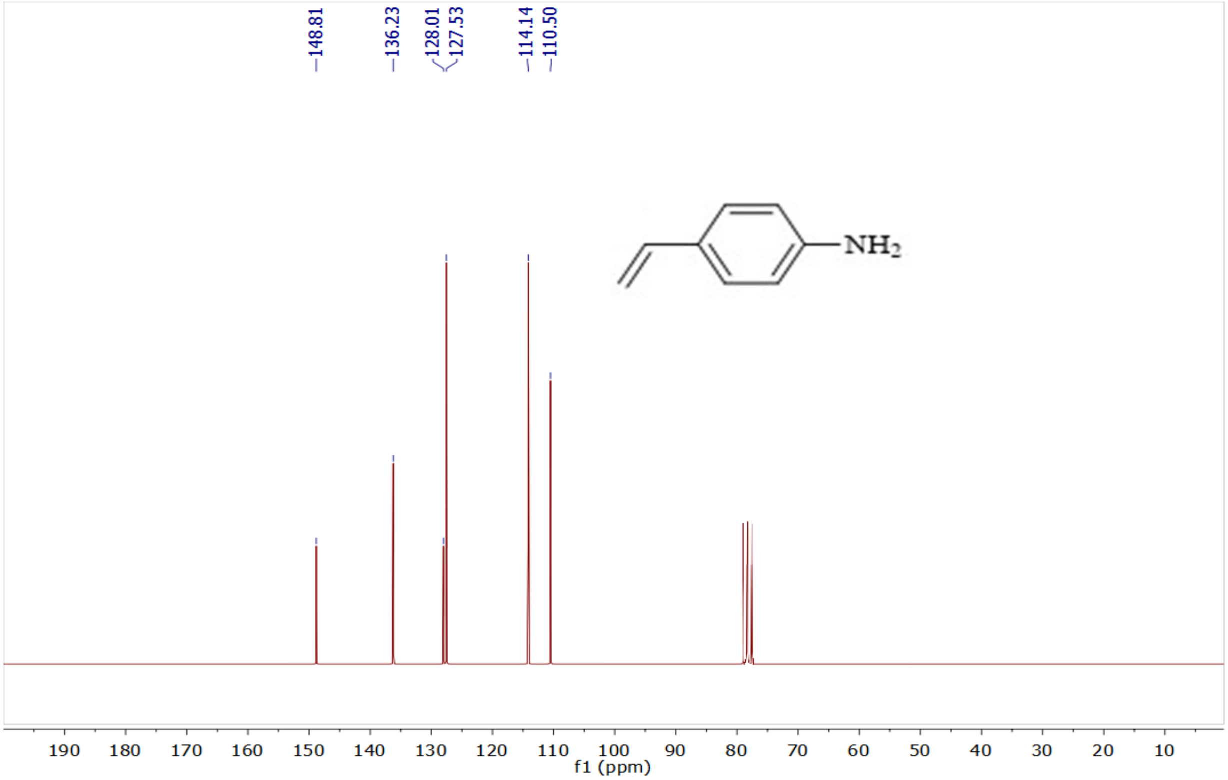

Fig. S12 ^{13}CNMR spectrum of d_2 (75 MHz, $CDCl_3$) (Table 6)

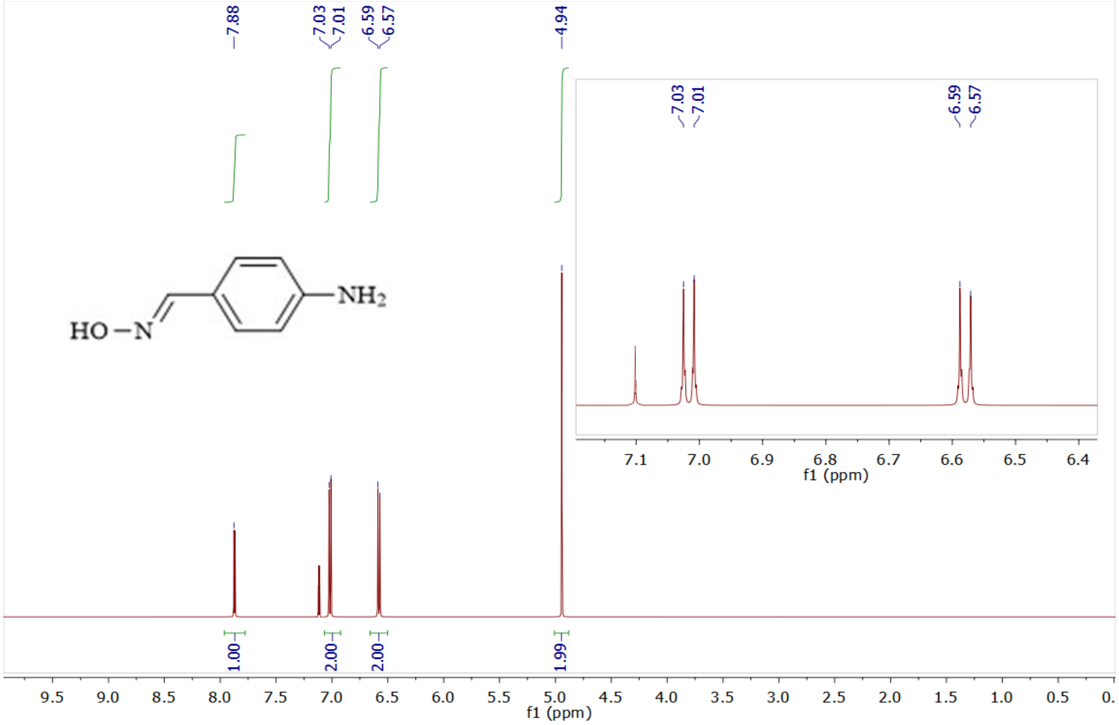

Fig. S13 ^{1}HNMR spectrum of e_1 (300 MHz, $CDCl_3$) (Table 6)

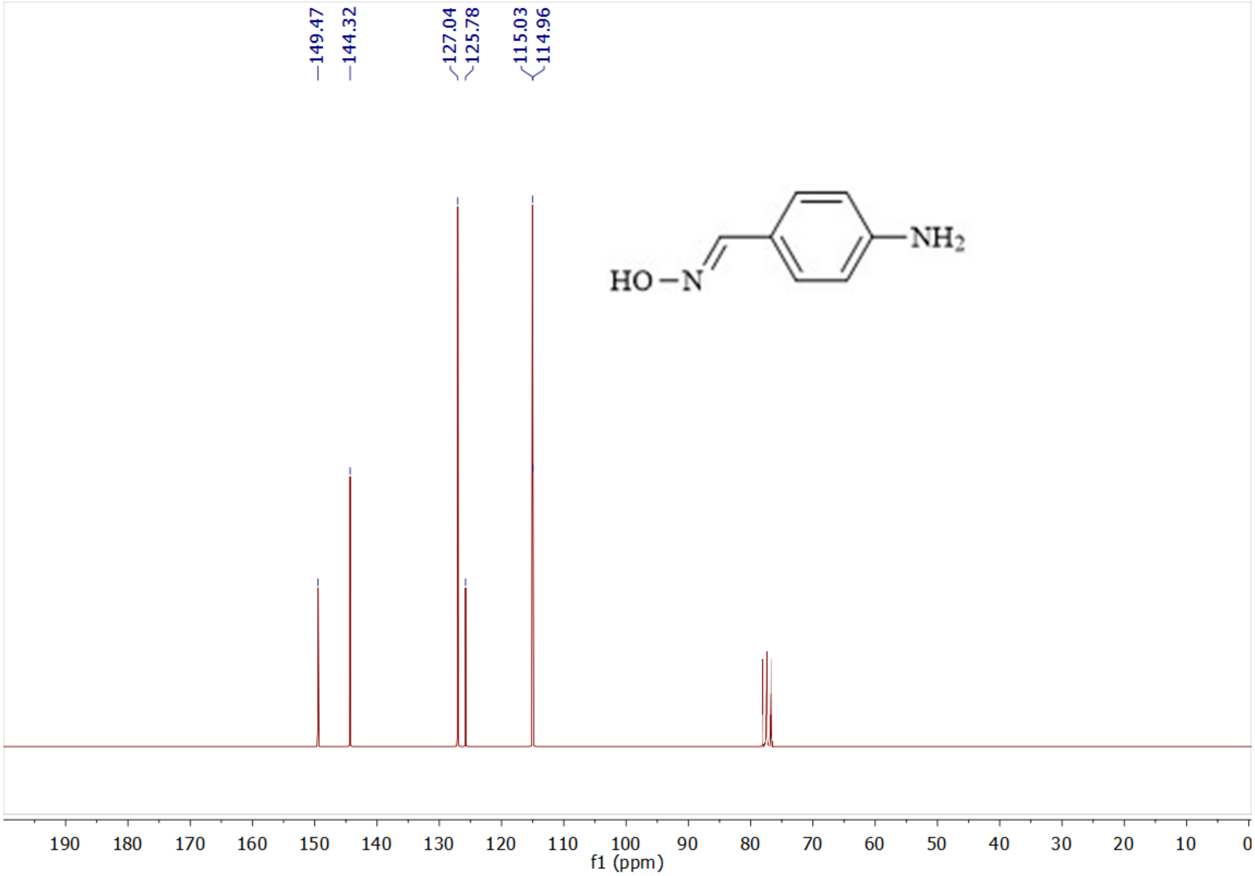

Fig. S14 ^{13}CNMR spectrum of e_1 (75 MHz, $CDCl_3$) (Table 6)

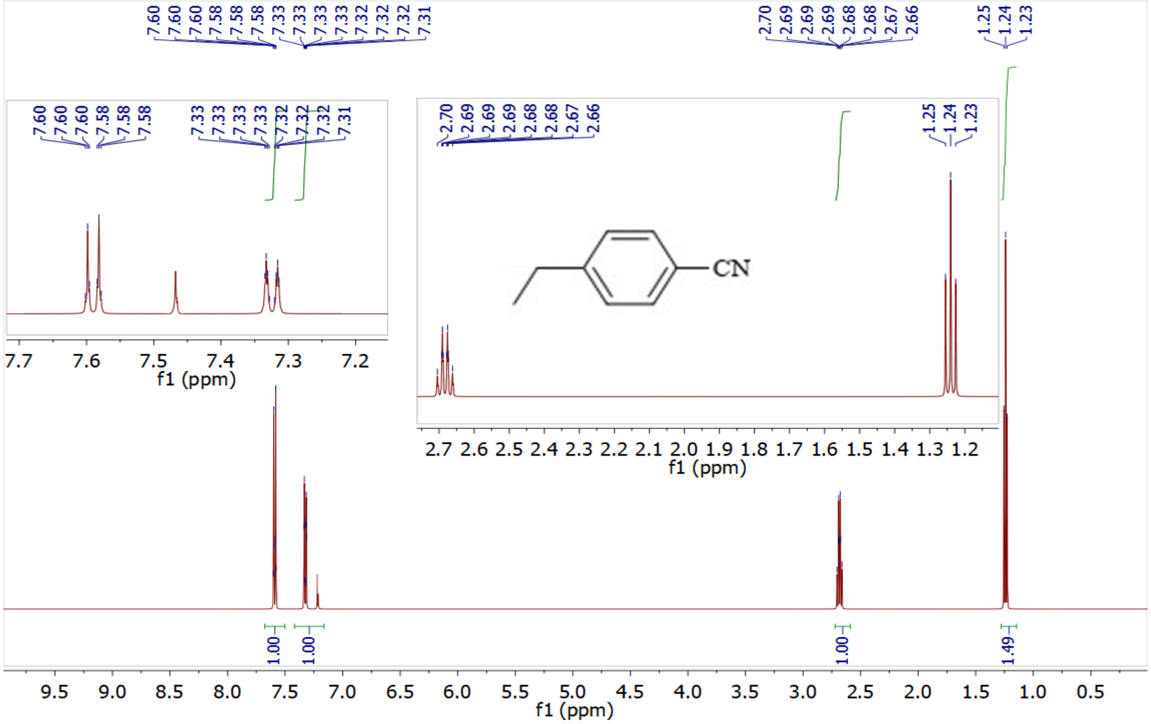

Fig. S15 ^{1}HNMR spectrum of g_1 (300 MHz, $CDCl_3$) (Table 6)

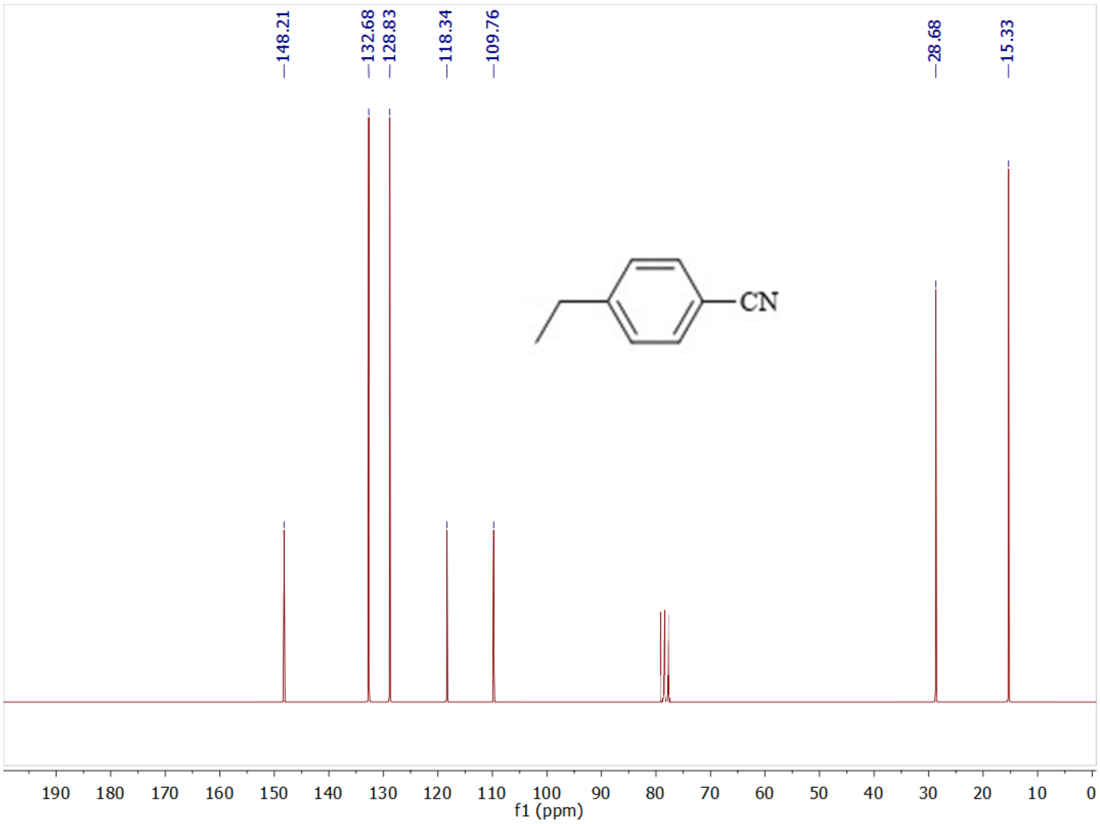

Fig. S16 ^{13}CNMR spectrum of g_1 (75 MHz, $CDCl_3$) (Table 6)

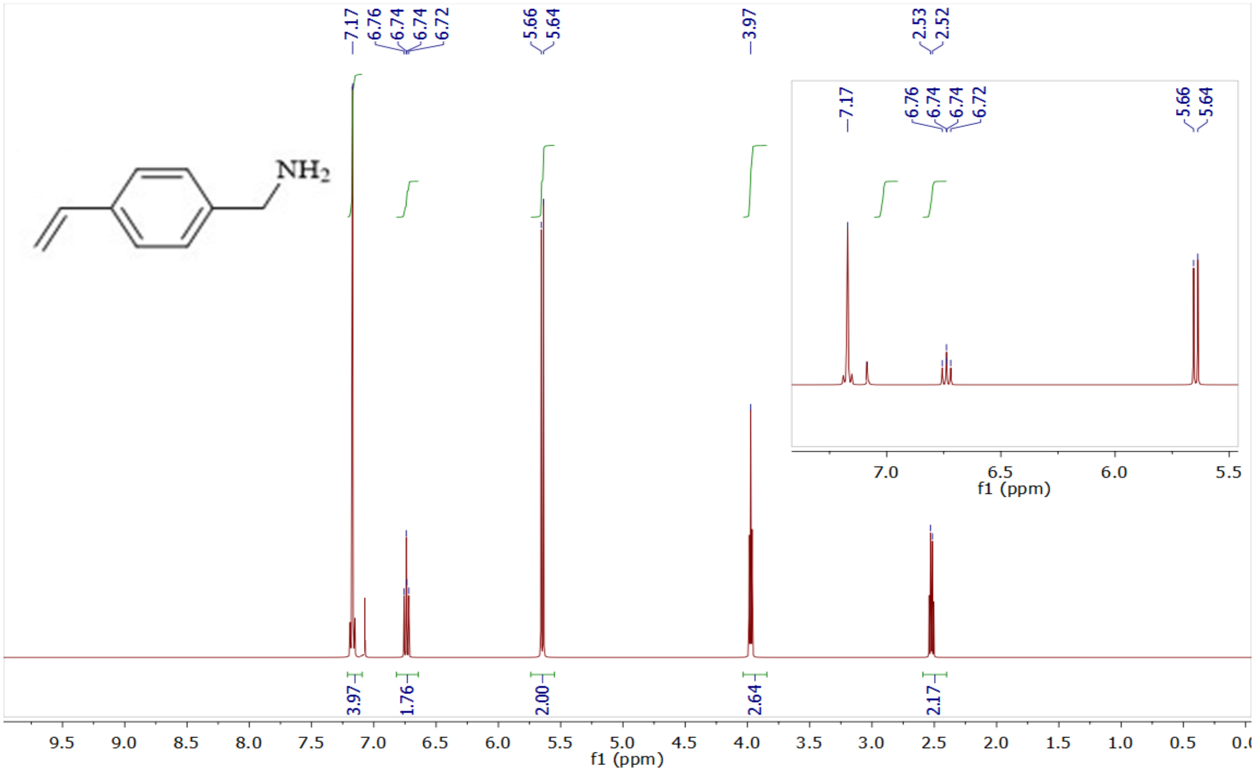

Fig. S17 ^{1}HNMR spectrum of g_2 (300 MHz, $CDCl_3$) (Table 6)

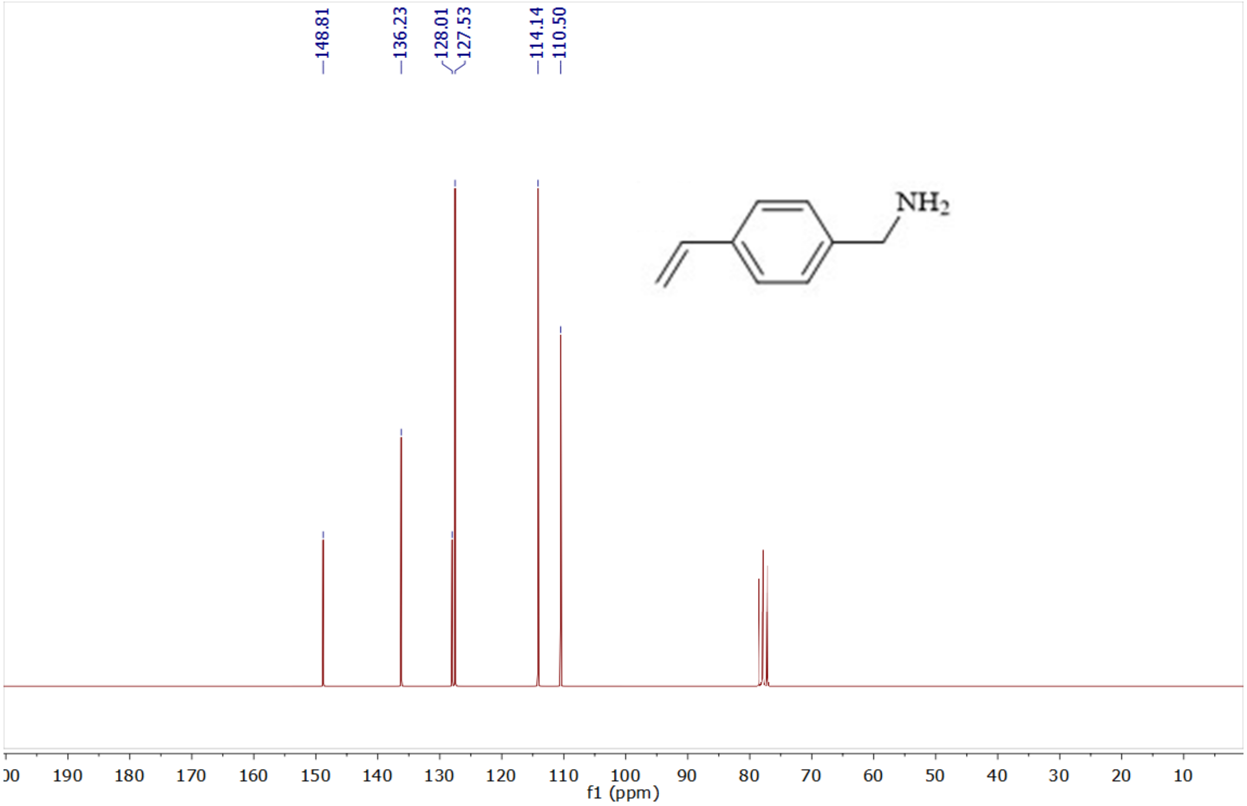

Fig. S18 ^{13}CNMR spectrum of g_2 (75 MHz, $CDCl_3$) (Table 6)

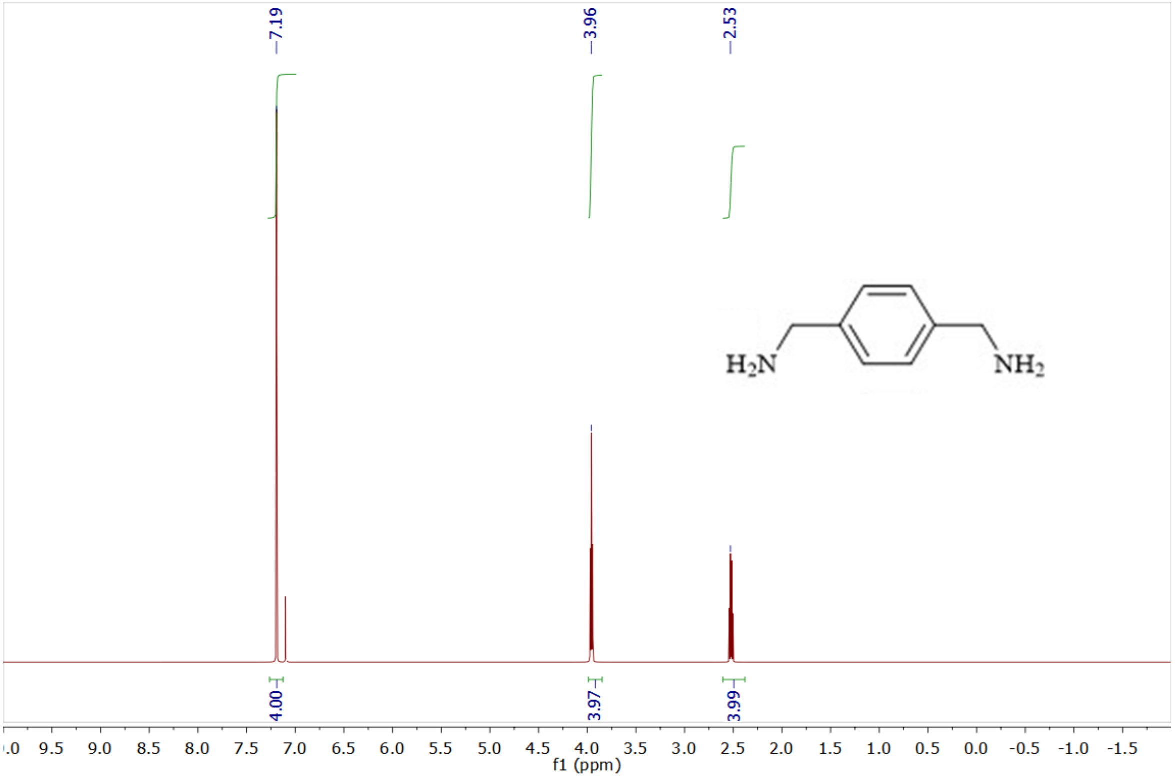

Fig. S19 ^{1}HNMR spectrum of h_2 (300 MHz, $CDCl_3$) (Table 6)

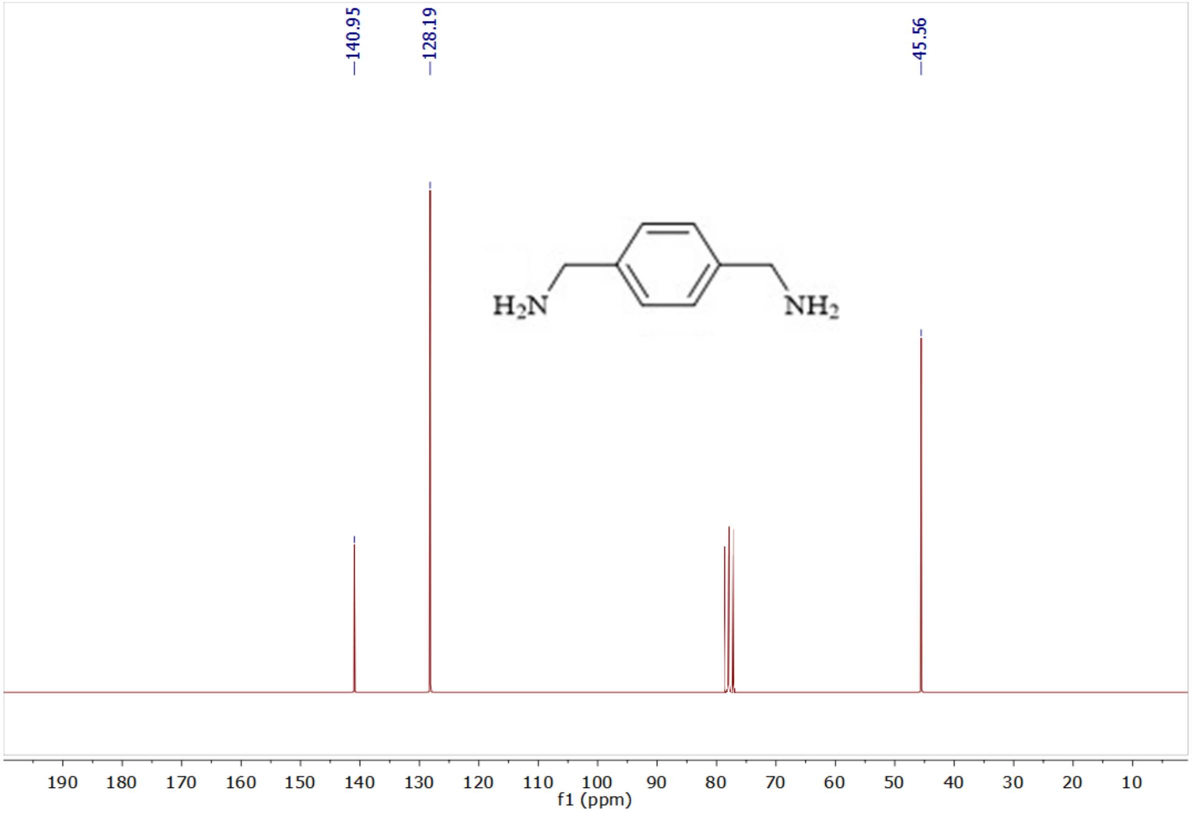

Fig. S20 ^{13}CNMR spectrum of h_2 (75 MHz, $CDCl_3$) (Table 6)

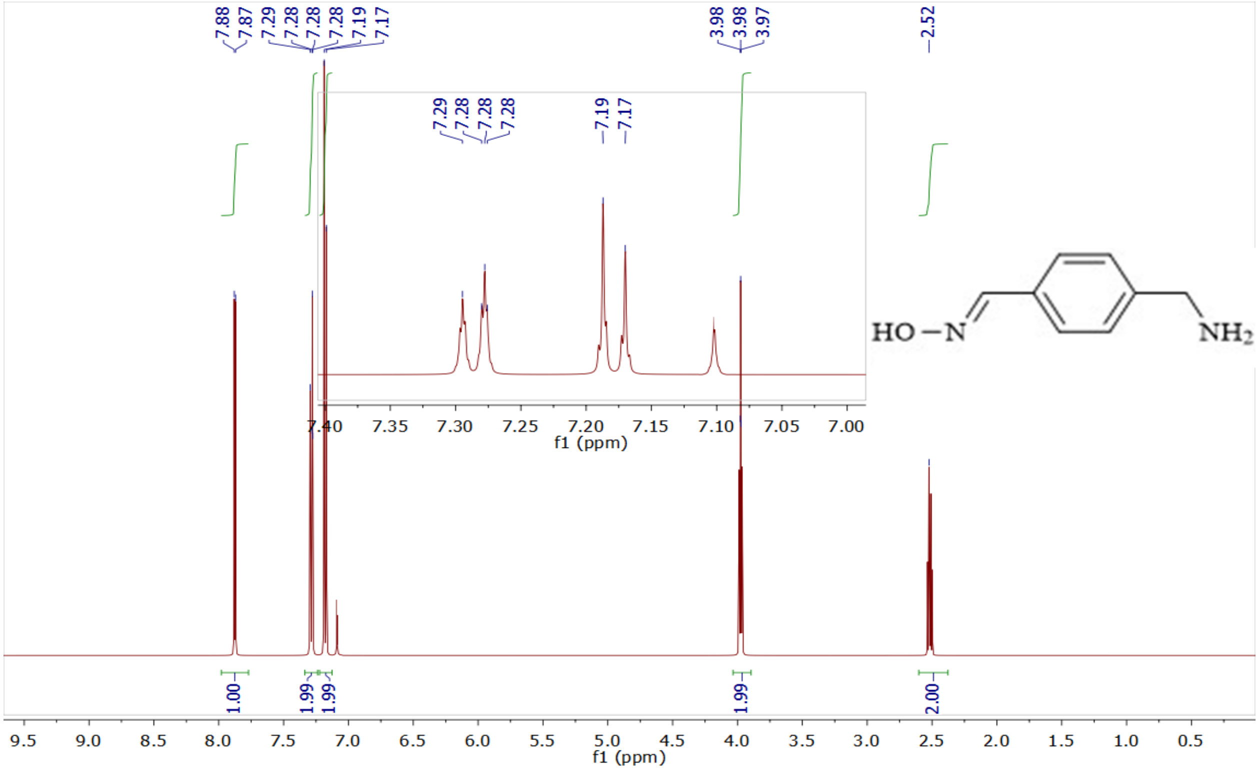

Fig. S21 ^{1}HNMR spectrum of h_3 (300 MHz, $CDCl_3$) (Table 6)

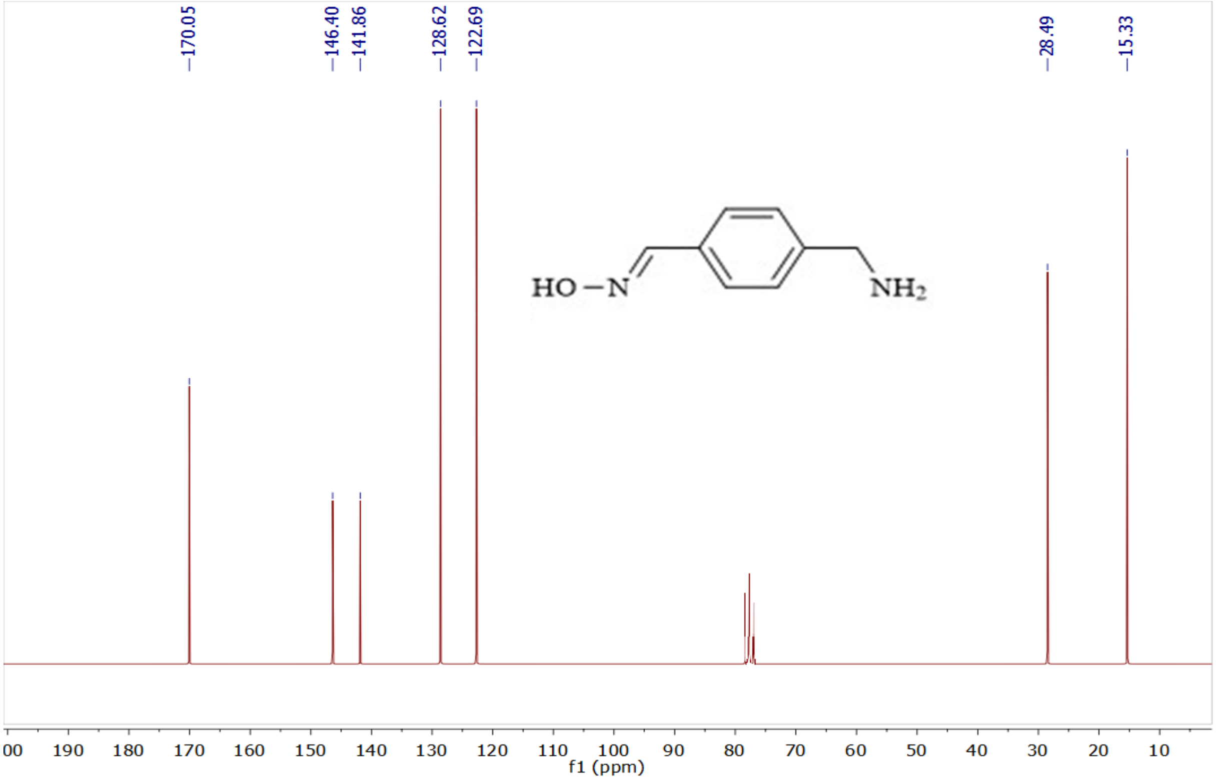

Fig. S22 ^{13}CNMR spectrum of h_3 (75 MHz, $CDCl_3$) (Table 6)

References

[1] D.W. Sheibley, L.L. Rieker, L.C. Hsu and M.A. Manzo, U.S. Pat., 1982, 4,357,402.

[2] Z. Wang, J. Luo, X.X. Zhu, S. Jin and M.J. Tomaszewski, Functionalized cross-linked poly (vinyl alcohol) resins as reaction scavengers and as supports for solid-phase organic synthesis. J. Comb. Chem., 2004, 6, 961-966.

[3] Y. Wan, W. Huang, Z. Wang and X.X. Zhu, Preparation and characterization of high loading porous crosslinked poly (vinyl alcohol) resins. Polymer, 2004, 45, 71-77.

[4] T. Nishikata, H. Tsutsumi, L. Gao, K. Kojima, K. Chikama and H. Nagashima, Adhesive catalyst immobilization of palladium nanoparticles on cotton and filter Paper: applications to reusable catalysts for sequential catalytic reactions. Adv. Synth. Catal., 2014, 356, 951-960.
